# A comparison of microcrystal electron diffraction and X-ray powder diffraction for the structural analysis of metal–organic frameworks

**DOI:** 10.1107/S1600576724012068

**Published:** 2025-02-28

**Authors:** Erik Biehler, Silvina Pagola, Daniel Stam, Johannes Merkelbach, Christian Jandl, Tarek M. Abdel-Fattah

**Affiliations:** a Applied Research Center, Thomas Jefferson National Accelerator Facility, Department of Molecular Biology and Chemistry Christopher Newport University Newport News VA23606 USA; b Department of Chemistry and Biochemistry Old Dominion University 4501 Elkhorn Avenue Norfolk VA23529 USA; c ELDICO Scientific AG, 5234Villigen, Switzerland; SLAC National Accelerator Laboratory, Menlo Park, USA

**Keywords:** MicroED, X-ray powder diffraction, metal–organic frameworks, green chemistry

## Abstract

This study successfully implemented microcrystal electron diffraction (microED) and X-ray powder diffraction (XRPD) for the crystal structure determination of a new phase, TAF-CNU-1, Ni(C_8_H_4_O_4_)·3H_2_O, solved by microED from single microcrystals in the powder and refined at the kinematic and dynamic electron diffraction theory levels. This nickel metal–organic framework (MOF), together with its cobalt and manganese analogues with formula *M*(C_8_H_4_O_4_)·2H_2_O with *M* = Mn^II^ or Co^II^, were synthesized in aqueous media as one-pot preparations from the corresponding hydrated metal chlorides and sodium terephthalate, as a promising ‘green’ synthetic route to moisture-stable MOFs. The crystal structures of the two latter materials have been previously determined *ab initio* from X-ray powder diffraction. The advantages and disadvantages of both structural characterization techniques are briefly summarized. Additional solid-state property characterization was carried out using thermogravimetric analysis, scanning electron microscopy and Fourier transform infrared spectroscopy.

## Abstract 2

Microcrystal electron diffraction (microED) was used to determine the crystal structure of a recently synthesized metal–organic framework (MOF) from powder crystallites. The structural descriptions of two other MOFs by microED are compared with results previously published using X-ray powder diffraction supported by density functional theory calculations.

## 1. Introduction

Porous crystalline solids such as metal–organic frameworks (MOFs), synthesized for the first time by Yaghi’s group (Li *et al.*, 1999 ▸), have gained a lot of attention in crystal engineering and related disciplines due to their potential myriad applications in catalysis, gas adsorption and separation, pharmaceutical drug delivery, molecular recognition processes, harmful substance storage (Gándara & Bennett, 2014 ▸), green chemistry *etc*. MOFs are easy to synthesize and several synthetic methods are available, such as solvothermal, mechano­chemical, microwave-assisted, electrochemical and others (Lee *et al.*, 2013 ▸). In addition, the properties of MOFs are tunable and amenable to rational design by the selection of appropriate metal ions at the nodes. The interplay of their physicochemical properties (including their oxidation states and catalytic properties) and those of the organic linkers leads to a large diversity of possible crystal-engineered MOF topologies, porosities, surface areas *etc.* that can be tailored to perform sought-after functions.

While many synthetic methods for the preparation of MOFs are effective, a potential green approach relies on water-based processes, which simply refers to MOF synthesis using water as the reaction medium. Water-based syntheses offer the advantage of being safe and cost effective, reducing the use of harmful organic solvents. These often require conditioning (degassing, drying *etc.*) before the reactions and must be disposed of after use, leading to increased waste and energy consumption. Water-based syntheses can afford sustainable chemistry, allowing the large-scale production of MOFs with reduced environmental impact, avoiding the use of hazardous organic solvents. Since water-based syntheses can lead to chemically stable MOFs under atmospheric moisture conditions, in some cases water-based syntheses could prove additionally advantageous over other synthetic methods.

Our team has explored the application of precious metals like gold, silver, platinum and palladium and their ability to catalyze the hydrolysis of sodium borohydride, NaBH_4_ (Huff *et al.*, 2018 ▸; Huff, Dushatinski & Abdel-Fattah, 2017 ▸; Huff, Long *et al.*, 2017 ▸). The scarcity and cost of using precious metals as catalysts have led many researchers to investigate effective and cheaper alternatives using more abundant elements. Transition metals such as cobalt, manganese and nickel have been studied for their catalytic abilities and their relatively low cost (Udani & Ronning, 2015 ▸; Ma *et al.*, 2016 ▸). Cobalt has been shown to be a stable catalyst for water splitting reactions, as well as being able to improve the electrode’s performance and kinetically enhance certain reactions (Zidki *et al.*, 2012 ▸; Feizi *et al.*, 2019 ▸). Manganese has found uses as a catalyst in hydrogenation and de­hydrogenation reactions, organic syntheses, and oxidation reactions (Widegren *et al.*, 2017 ▸; Kallmeier & Kempe, 2017 ▸; Carney *et al.*, 2016 ▸). Similarly, nickel has been explored for its use in fuel cells, methane reformation, water splitting and other important energetically efficient green processes (Sun *et al.*, 2005 ▸; Xu *et al.*, 2001 ▸; Bulakhe *et al.*, 2013 ▸; Netskina *et al.*, 2021 ▸).

Purified terephthalic acid is the preferred raw material for the manufacture of polyesters (Kaduk, 2000 ▸), such as polyethyl­ene terephthalate (PET), used in a wide variety of consumer and industrial products, such as fibres for clothing and rigid or flexible plastic containers. The crystal structures of various terephthalate salts have been solved from synchrotron X-ray powder diffraction (XRPD) (Kaduk, 2002 ▸). These materials can be encountered as residues in industrial manufacturing processes that use terephthalic acid (Kaduk, 2000 ▸), although typically as mixtures rather than single phases. In addition, the crystal structures of the isomorphous zinc, cobalt and nickel basic salts of the doubly deprotonated terephthalate ion with general formula *M*_2_(C_8_H_4_O_4_)(OH)_2_ with *M* = Zn^II^, Co^II^ or Ni^II^ have been reported (Markun *et al.*, 2022 ▸). The use of sodium terephthalate as an electrode material in sodium-ion batteries has also been studied (Park *et al.*, 2012 ▸; Wang *et al.*, 2020 ▸).

The present work reports room-temperature water-based MOF syntheses combining Ni^II^, Co^II^ or Mn^II^ ions with terephthalic acid as the organic ligand. This environmentally friendly method potentially affords the large-scale preparation of these materials for uses in environmental remediation, energy storage, catalysis, sensors or other applications. With the aim of structurally characterizing these materials, our work implemented microcrystal electron diffraction (microED), a decade-old technique for the structural analysis of solids from individual single crystallites of micrometre or even nanometre sizes (Hattne *et al.*, 2015 ▸). MicroED evolved from cryo-EM methods used in structural biology (Yang *et al.*, 2021 ▸), building on advances such as the continuous rotation method, sensitive direct electron detectors, innovations in electron optics, and new software and data analysis algorithms (Nannenga *et al.*, 2018 ▸). Although microED was also initially developed for structural biology and first demonstrated with the structure of lysozyme (Shi *et al.*, 2013 ▸), other successfully determined structures include other proteins (Hattne *et al.*, 2015 ▸; Rodriguez, 2015 ▸), protein–ligand complexes (Clabbers *et al.*, 2020 ▸), crystalline polymers (Anderson *et al.*, 2021 ▸), supramolecular organic frameworks (Marchetti *et al.*, 2023 ▸), small organic molecules (Jones *et al.*, 2018 ▸; Yang *et al.*, 2021 ▸), pharmaceuticals (Ge *et al.*, 2024 ▸; Gogoi *et al.*, 2023 ▸), natural products (Delgadillo *et al.*, 2024 ▸), reactive and even seemingly amorphous organometallics (Jones *et al.*, 2019 ▸), MOFs (Sala *et al.*, 2024 ▸; Aykanat *et al.*, 2021 ▸), inorganic materials, and radioactive minerals and quickly decaying materials (Saha *et al.*, 2022 ▸). Processes such as high-throughput screening of natural products and organic molecular solids of pharmaceutical interest, as well as studies of their impurities and polymorphism, can be drastically accelerated. Other materials such as mechanochemical products which do not grow as single crystals have been structurally characterized (Sala *et al.*, 2024 ▸).

Theoretical aspects and a brief historical background of microED are described by Saha *et al.* (2022 ▸). Basically, electron diffraction has been known since the late 1920s. However, due to dynamic effects in electron diffraction data as a result of multiple diffraction events, the diffracted intensities are not directly proportional to the amplitude squared of the structure factors, so they need corrections which at that time were not straightforward to do. The availability of larger acceleration voltages nowadays reduces the frequency of such events, facilitating data analysis (Saha *et al.*, 2022 ▸). Most importantly, microED has unique advantages, such as (i) the dramatic reduction in the crystal size (of any type of material) for which structural analysis at atomic resolution becomes possible in comparison with X-ray single-crystal diffraction, (ii) experimental access to the three-dimensional reciprocal lattice, H-atom positions and absolute stereochemistry, in contrast to X-ray powder diffraction, (iii) the relatively short data collection times (without the need for crystal growth time), (iv) the small amounts of analyte used after minimal purification processes, and (v) the increasing availability of dedicated electron diffractometers, in comparison with necessarily large (and expensive to build) experimental facilities, such as free-electron lasers, neutron diffractometers or synchrotrons. All of these advantages make this technique a breakthrough in crystallography, complementing traditional X-ray and neutron diffraction methods. However, some drawbacks remain, such as beam damage to the material (even though the doses are relatively low), preferred orientation of the crystallites, higher residuals compared with single-crystal X-ray diffraction, particularly for refinements at the kinematic level (possibly involving less accurate crystallographic results), and the possible decomposition of the material under high vacuum (*e.g.* the release of water or solvents).

MicroED data collection and analysis have been detailed by Hattne *et al.* (2015 ▸) and Saha *et al.* (2022 ▸). Our work describes these processes in Section 2.3 and in the supporting information. A large fraction of this article is dedicated to comparing the quality of the crystallographic parameters determined from XRPD and microED for the three MOFs synthesized. MicroED afforded the crystal structure of a new material, Ni(C_8_H_4_O_4_)·3H_2_O (**I**), also called TAF-CNU-1, refined at the dynamic electron diffraction theory level. The MOFs containing Co^II^ and Mn^II^, labelled **II** and **III**, respectively, were identified by microED and XRPD, although their crystal structures are known (Kaduk, 2002 ▸). Thus, the least-squares refinements were kept at the kinematic level. The advantages and disadvantages that laboratory XRPD and microED encountered during phase identification and crystal structure determination procedures will be summarized. Additionally, scanning electron microscopy (SEM) was used for the examination of the morphology and particle sizes of all three materials, which were also characterized by Fourier transform infrared (FT-IR) spectroscopy and thermogravimetry.

## 2. Experimental

### 2.1. Materials and synthesis

All analytical grade chemicals (+99% purity) were purchased from Sigma–Aldrich (unless otherwise specified) and used as received. The MOFs reported herein were synthesized in aqueous solutions at room temperature in all cases, prepared with deionized water of 18 MΩ electrical resistance. The nickel precursor, nickel(II) chloride hexa­hydrate, NiCl_2_·6H_2_O (Alfa Aesar, 99% purity), was combined with the sodium salt of the linker, *i.e.* sodium terephthalate (Na_2_C_8_H_4_O_4_), herein labelled Na_2_BDC, in a 2:1 metal-to-linker molar ratio (Zhan & Zeng, 2016 ▸). Thus, 40 mmol of Na_2_BDC were dissolved in 50 ml of deionized water and this solution was added dropwise to an aqueous solution of nickel(II) chloride (80 mmol of NiCl_2_·6H_2_O dissolved in 100 ml of deionized water). The resulting solution was covered and stirred continuously for seven days under ambient conditions. After this, the product was centrifuged at 10000 rpm and the precipitate was collected and rinsed with deionized water to remove any leftover unreacted linker or ions. The powders obtained were pale green in colour. Finally, they were heated at 110 °C to remove any remaining moisture.

Identical synthetic procedures were used for the preparation of **II** and **III**. In these reactions, the metal precursors were cobalt(II) chloride hexahydrate (CoCl_2_·6H_2_O, Alfa Aesar, 99% purity) and manganese(II) chloride tetrahydrate (MnCl_2_·4H_2_O), respectively. Compound **II** was recovered as a pink powder and **III** as a yellowish light-brown powder.

### 2.2. X-ray powder diffraction (XRPD)

XRPD patterns were collected for crystal phase identification using a Bruker D2 Phaser X-ray powder diffractometer with Cu *K*α radiation (λ = 1.5418 Å), equipped with a LynxEye position-sensitive detector in Bragg–Brentano reflection optics and θ–θ configuration. The samples were mounted on 1.5 cm diameter × 0.5 mm depth zero-background silicon sample holders and spun at 15 rpm during data collection. A 1 mm air scattering screen was used. The X-ray energy range of the detector was electronically adjusted to avoid measuring fluorescent X-rays.

For TAF-CNU-1, an additional XRPD dataset was collected for use in crystal structure determination. This was done from 2θ = 2° up to 130° in steps of 0.02°. A counting time of 8 s per step and a position-sensitive detector opening of 1° were used in the 2°–60° 2θ interval, while 15 s per step counting time and a 3 mm air scattering screen were used in the 56°–130° 2θ interval. The two datasets were merged into one using the *DIFFRAC.EVA* software (Version 7.0.0.6; Bruker AXS GmbH, Karlsruhe, Germany). The software *EXPO2013* (Altomare *et al.*, 2013 ▸) was used for indexing and space group symmetry determination, and for the application of the direct methods (Hauptman & Karle, 1953 ▸) adapted to the analysis of XRPD data. Le Bail fits (Le Bail, 2005 ▸) were done with the software *GSAS* (Larson & Von Dreele, 2004 ▸) or *FULLPROF* (Rodríguez-Carvajal, 1993 ▸) implemented in *WinPLOTR* (Roisnel & Rodríguez-Carvajal, 2002 ▸).

### 2.3. Microcrystal electron diffraction (microED)

MicroED measurements were carried out at ELDICO Scientific Ltd as collaborative work. MicroED data were collected using an ELDICO ED-1 electron diffractometer at room temperature and the software *Eldix* (Version 1.4.2; ELDICO, 2023 ▸), a LaB_6_ source at an acceleration voltage of 160 kV (λ = 0.02851 Å), and a hybrid pixel detector (Dectris QUADRO). Powders of the materials studied were finely dispersed onto a standard transmission electron microscopy (TEM) grid (amorphous carbon on Cu). All measurements were carried out at room temperature. Suitable crystals were located and centred in scanning transmission electron microscopy (STEM) imaging mode, and electron diffraction data were recorded in continuous rotation mode. The last parts of the measurements showing significant beam damage were omitted from the data analysis.

Electron diffraction data were collected from crystallites of around 1–2 µm in size using a beam of 800 nm diameter. The data were analysed for crystal structure determination using the *APEX4* software (Bruker, 2022 ▸). Initial refinements of the three crystal structures (**I**, **II** and **III**) at the kinematic theoretical level were done with *SHELXL* (Sheldrick, 2015 ▸) in conjunction with *SHELXLE* (Hübschle *et al.*, 2011 ▸). Data for the dynamic refinement of TAF-CNU-1 (**I**) were processed using the *PETS2* software (Palatinus *et al.*, 2019 ▸). The dynamic refinement of TAF-CNU-1 was performed using *JANA2020* (Petříček *et al.*, 2014 ▸) starting from the crystal structure obtained by the kinematic refinement as the initial model. Geometric parameters of the dynamically refined structure were generated with the software *PLATON* (Spek, 2020 ▸). All three crystal structures were graphically represented using the software *Mercury* (Version 2023.2.0; Macrae *et al.*, 2020 ▸). Additional details of the microED data analysis can be found in Section S2 of the supporting information.

### 2.4. Thermogravimetric analysis (TGA)

For **I** (TAF-CNU-1), the thermogravimetric mass loss and its derivative were measured in a TA Instruments Q5000 balance using the high-resolution dynamic mode from room temperature to 500 °C, using a 2 °C min^−1^ heating rate, with resolution and sensitivity parameters 4 and 1, respectively. The N_2_ gas flow was 50 ml min^−1^ and 50 µl Pt pans were used. The thermogravimetric data of **II** and **III** were similarly collected in the interval from room temperature to 900 °C using a NETZSCH TG 209 F3 thermogravimetric balance.

### 2.5. FT-IR spectroscopy

FT-IR spectroscopy data were measured in a Shimadzu IR Tracer 100 spectrometer (Kyoto, Japan), equipped with an attenuated total reflectance (ATR) attachment (Shimadzu QATR-S, Kyoto, Japan). The datasets were used to confirm the functional groups in the materials and infer hydrogen-bonding features. Each sample was scanned in the 500–4000 cm^−1^ wavenumber interval, using 24 scans and 0.25 cm^−1^ resolution. Data analysis was performed using the *SpectraGryph* software (Version 1.2.15; Mengues, 2020 ▸).

### 2.6. Scanning electron microscopy (SEM)

SEM images were collected using a JEOL JSM-6060LV instrument coupled with a ThermoFisher Scientific UltraDry energy-dispersive X-ray detector (EDS) for elemental analysis and mapping of the materials synthesized, yielding particle sizes and morphologies.

## 3. Results and discussion

### 3.1. Crystal phase identification and crystal structure determination

Laboratory XRPD data were used for the identification of crystalline phases in all three reaction products, with the objective of determining the crystal structures of the new materials. The analysis included the consideration of peak position changes due to expected differences in lattice parameters, plus possible preferred orientation effects, and the existence of impurity peaks. This analysis led us to suspect that there were two possibly isostructural compounds, **II** and **III**, because of the overall similarities in their XRPD data. An overlay of these datasets is shown in Fig. S1.

None of the unit cells found with *EXPO2013* (Altomare *et al.*, 2013 ▸) indexed all the peaks of **II** or **III**, pointing to the presence of impurities leading to some weak peaks. In contrast, the XRPD data of TAF-CNU-1 (**I**) were indexed with two different sets of lattice parameters, with unit-cell volumes in an approximately 2:1 ratio and a common *b* axis of ∼22.99 Å, suggesting a symmetry relationship between them. These results were a monoclinic structure with *a* = 13.6567 Å, *b* = 22.9964 Å, *c* = 4.5895 Å, α = 90.00°, β = 92.4557°, γ = 90.00° and unit-cell volume *V* = 1430 Å^3^, with an indexing figure of merit (*M*) of 9 and space group *P*2_1_/*a*; and an orthorhombic structure with *a* = 20.451 Å, *b* = 22.9816 Å, *c* = 6.1889 Å, α = 90.00°, β = 90.00°, γ = 90.00°, *M* = 8 and *V* = 2897 Å^3^. The space group candidate for the second structure was *Pbnn*, although crystal structure determination in all space groups compatible with the systematic absences was necessary to confirm it as correct (or not).

In a subsequent *EXPO2013* run starting the space group search from the orthorhombic unit cell above and the formula NiC_8_H_10_O_7_, the possible space groups were *Pbnm* and *Pbn*2_1_ (with the highest figure of merit 0.168), followed by *Pbnn* with figure of merit 0.103. The remaining space groups had considerably lower figures of merit, although the first one in that section was *Pbcn* (later determined as correct), with figure of merit 0.037. The search among all possible space groups was further complicated by the likely presence of impurity peaks (as in **II** and **III**). The two above unit-cell candidates were evaluated as possibly correct using the Le Bail method. The fits and their agreement factors are shown in Figs. S2 and S3, respectively. However, since the presence of impurities was possible and their peak positions were unknown, the results of the space group symmetry search were inconclusive.

SEM images (see Section 3.6) did not show needle or plate particle morphologies. Hence, the presence of preferred orientation effects in the laboratory XRPD data is not immediately obvious, although it cannot be ruled out either. Since crystal structure determination using *EXPO2013* was unsuccessful, at this stage it seemed necessary to obtain better diffraction data, for example high-resolution (synchrotron) XRPD data up to *d* < 1 Å (for crystal structure determination), in which preferred orientation would be substantially reduced (or essentially avoided) due to the use of transmission optics from a capillary containing the powder, spun during data collection in Debye–Scherrer configuration. However, this was unnecessary since the crystal structures could be solved by microED.

**Figure 1:**
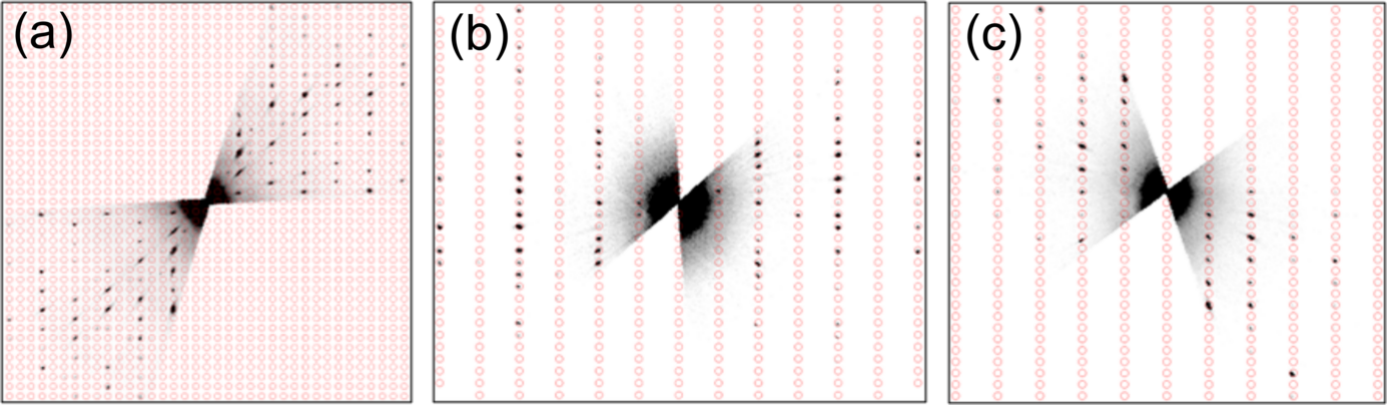
Reconstructed reciprocal-space images of one of three microED data sets collected from TAF-CNU-1 (**I**). The calculated positions of expected reflections using the orthorhombic unit-cell parameters (see Section 3.3) are represented by red circles for the orientations (*a*) 0*kl*, (*b*) *h*0*l* and (*c*) *hk*0.

MicroED experimental details are described above in Section 2.3 and in Section S2 of the supporting information. Initial models were calculated within one hour of work (for each of **I**, **II** and **III**). Fig. 1 ▸ shows microED data for TAF-CNU-1.

### 3.2. Crystal structure analysis of II and III by microED (kinematic diffraction approach)

The crystal structures of **II** and **III** have been already solved from XRPD data (Kaduk, 2002 ▸). These two materials are also isostructural with *M*(C_8_H_4_O_4_)·2H_2_O, *M* = Fe^II^, Mg^II^. Their crystallographic descriptions by microED (this work) are reported in Table 1 ▸. Note that these hydrates are stable in the high-vacuum conditions used for microED data collection.

**Figure 2:**
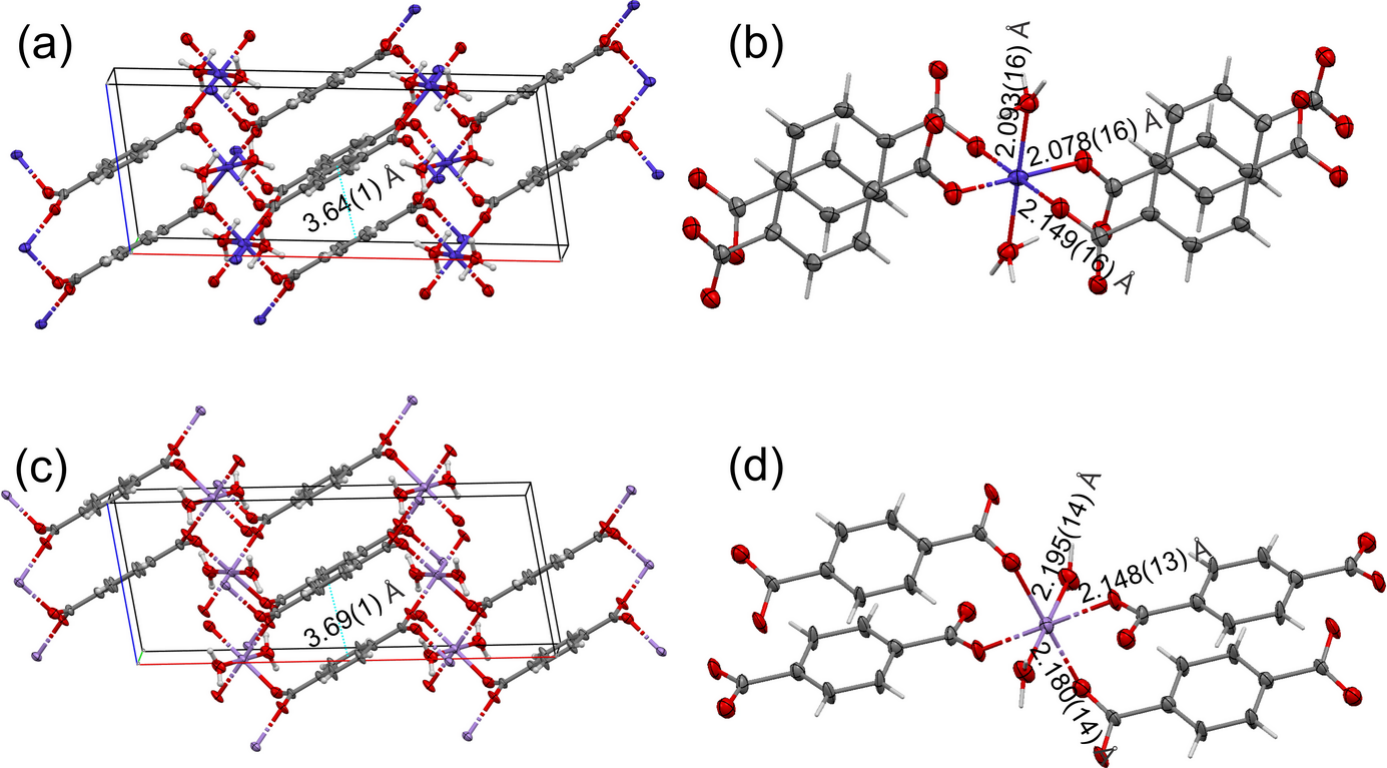
(*a*) A view of the crystal structure of **II** obtained by microED (kinematic refinement) approximately along the *b*-axis direction, represented using *Mercury* (Version 2023.2.0; Macrae *et al.*, 2020 ▸). Layers of octahedrally coordinated metal cations alternate with terephthalate layers. The distance between aromatic terephthalate ring centroids is shown (the standard uncertainty was estimated by the authors). C atoms are shown in grey and O atoms in red, while H atoms have been omitted for clarity. (*b*) The coordination sphere around Co^II^ (in blue), showing the three crystallographically independent Co—O distances (H atoms shown as light-grey sticks). (*c*) The crystal structure of **III** from microED (kinematic refinement), also viewed approximately along the *b* axis. The distance between aromatic ring centroids (with standard uncertainty estimated by the authors) is shown. Mn atoms are shown in violet, while H atoms have been omitted for clarity. (*d*) The coordination sphere around Mn^II^, showing the three crystallographically independent Mn—O distances (H atoms shown as light-grey sticks). All atoms except H are shown as 50% probability displacement ellipsoids.

The crystal structures of **II** and **III** (Kaduk, 2002 ▸) are made of alternating layers of octahedrally coordinated metal cations and terephthalate anions (Fig. 2 ▸). The octahedra are isolated. The metal–oxygen bonding in the magnesium compound (not studied in this work) is essentially ionic, while for the remaining metal centres (including Co and Mn) the coordination bonds show significant covalent character. Four terephthalate O atoms are equatorially coordinated to the metal cations and two water molecules occupy the axial positions. Fig. 2 ▸ shows the crystal packing in **II** and **III** as determined by microED (at the kinematic diffraction theory level) and details of the coordination sphere around the metal cations.

Considering the experimentally determined formula (including H-atom positions), the assigned oxidation state in each of the cations is +2, since both carboxylate groups of the tereph­thalates are deprotonated. Thus, it is expected that the two carbon–oxygen bond distances will be very similar, due to the presence of a negative charge delocalized in the two carbon–oxygen bonds, rather than long and short distances corresponding to a single C—O and a double C=O bond, respectively. In **II**, there are two crystallographically independent carbon–oxygen distances in the only carboxylate group of the asymmetric unit, which are 1.23 (2) and 1.29 (2) Å (0.06 Å difference) by laboratory XRPD, as calculated using *PLATON* (Spek, 2020 ▸) from the published Crystallographic Information Framework (CIF) file (Kaduk, 2002 ▸). In our microED results, those distances are 1.245 (16) and 1.271 (16) Å (0.026 Å difference). The reported distances optimized by density functional theory (DFT) (Kaduk, 2002 ▸) using *CASTEP* (Clark *et al.*, 2005 ▸) are 1.289 and 1.272 Å (0.017 Å difference). The smaller difference in the carbon–oxygen distances calculated by DFT (in agreement with a delocalized negative charge in the carboxylate anion) is closer to the microED results, while the XRPD results suggest a double and a single bond. This result indicates that microED (even at the kinematic level) affords accurate chemical bonding details (such as distinguishing carbon–oxygen bond order), which may not be obvious from the XRPD data. In the analysis of the latter, one must choose values for bond length restraints and their weights during Rietveld fits, at least partially imposing the chosen features on the structural model. Moreover, the calculation of the best structural model can involve carrying out several Rietveld fits for comparison purposes, a time-consuming task. Although crystal structure determination from powders is highly meritorious, the results are less accurate than those from single-crystal X-ray diffraction. This is chiefly due to the availability of a large number of independently measured structure factor amplitudes in the latter, and the fact that the three-dimensional reciprocal lattice and its symmetry are experimentally determined directly from a single crystal. From XRPD, supporting DFT optimizations (also time consuming and generally requiring access to high-performance computer systems) are very often used to confirm chemical bonding details and further validate crystal structure determinations from powders. This includes the calculation of H-atom positions and hydrogen-bonding motifs, which are generally not accessible from XRPD.

For **III**, the analogous carbon–oxygen distances are 1.259 (9) and 1.262 (8) Å by XRPD, as calculated from the published CIF file (Kaduk, 2002 ▸), 1.298 and 1.267 Å by *CASTEP*, and 1.258 (14) and 1.270 (15) Å by microED. The small differences (0.003, 0.031 and 0.012 Å, respectively) are in agreement with a delocalized negative charge between the two carboxylate O atoms, as expected. The distances between the tereph­thalate centroids in **II** are 3.64 (1) Å (microED) and 3.65 (1) Å (XRPD; Kaduk, 2002 ▸). For **III**, they are 3.69 (1) Å (microED) and 3.70 (1) Å (XRPD; Kaduk, 2002 ▸). Note that the quoted standard uncertainties were estimated by the authors, since *Mercury* (Version 2023.2.0) does not report them.

Table 2 ▸ compares other selected bond distances obtained by XRPD and microED. Note that the differences are both positive and negative. The absolute values of the relative percent differences, taking the XRPD results in the published CIF files (Kaduk, 2002 ▸) as the true values, are below 1% in four of the six distances shown for **II** and **III**, and below 2% in all six cases. If one considers the microED results as the true values instead, three of the six absolute values of the relative percent differences are less than 1%, and all are below 2%. Additionally for **III**, the trend in bond distance values that correspond to Mn—O_carboxylate_ and Mn—O_water_ is different in the XRPD and in the microED results. The longest Mn—O_water_ distance of 2.195 (14) Å by microED does not correspond to the longest Mn—O bond by XRPD [2.197 (5) Å], which is to a carboxylate O atom. The differences for the *CASTEP* distances are larger and will not be discussed here.

The unit-cell parameters are another important set of crystallographic results to compare, in order to improve our understanding of the advantages and disadvantages of using XRPD and microED for crystal structure determination. These are shown in Table 3 ▸. The refinements of the XRPD data collected in our laboratory were carried out starting from the lattice parameters determined by microED (*EXPO2013* did not find the unit-cell parameters of **II** and **III**). In the Le Bail fits shown in Figs. S5 and S6, the unit-cell parameters were refined together with the sample shift error (due to Bragg–Brentano optics in reflection geometry), and later the transparency error was added to the fit. Peak profile parameters were refined as needed. The two unit cells obtained by XRPD (Table 3 ▸) are very close within experimental error and are essentially equivalent down to the second decimal place. This is expected but also noteworthy, since the sample syntheses (even possibly the particle morphology) and sample mounting on the respective sample holders were slightly different. Since systematic errors such as sample shift vary from sample mounting to sample mounting, the similarity of the lattice parameters obtained by XRPD suggests that such systematic aberrations (and others which may shift the peak positions) have been reasonably well corrected in both XRPD datasets.

Regarding how the lattice parameters from XRPD compare with those from microED, it is readily seen that there is a discrepancy in the *a* unit-cell parameter of **II** (Table 3 ▸). We then investigated whether an acceptable XRPD fit could be obtained by starting another fit (also from the microED unit cell) but fixing the unit-cell values, assuming them to be accurate, and refining them only after the known XRPD aberrations that could modify peak positions, plus the peak shape parameters, had been refined. This refinement strategy led to several shifted peaks and higher agreement factors. Hence, the unit-cell parameters previously refined by XRPD (Kaduk, 2002 ▸) and those of this work are deemed more accurate than the unit cells refined by microED.

The accurate determination of unit-cell parameters is known to be a strength of the XRPD technique (laboratory or synchrotron) in comparison with X-ray single-crystal diffraction. This may be at least in part because a unique peak shape function with a set of refinable parameters is used to fit the complete XRPD dataset. Good physical models and algorithms are available in various Rietveld software packages to correct for the expected systematic errors that could also affect the peak positions. The addition of an internal standard would further increase the accuracy of the lattice parameters determined. In single-crystal X-ray diffraction instead, the spot profile parameters (and so the position of the reflection centroids) are typically less well determined, and they also depend on the regions of the area detector measuring the spots. In a typical fit of spot profiles using *APEX4* there may be frames with well determined profiles and others with a rather poor description for them, leading to a larger spread in the values of the reflection centroids with respect to their expected positions, and thus a smaller precision in the lattice parameters. Note also that the precision of the unit-cell and other crystallographic parameters from Rietveld fits may be considered overestimated [Scott (1983 ▸) and references therein], due to the error correlation in adjacent powder diffraction intensities and the presence of errors other than counting statistics in most data sets.

While in principle unit-cell parameters from microED and single-crystal X-ray diffraction should be comparable, microED experiments pose additional instrumental and sample-related difficulties. Instrumental aberrations, such as mechanical instabilities of the sample stage and distortion effects by the lens system of the measurement device, can be reduced using software (Brázda *et al.*, 2022 ▸) or by using instrumentation with no lenses between the sample and the detector (Simoncic *et al.*, 2023 ▸), although they may not be completely removed. Moreover, typically fewer reflections are gathered and used for unit-cell refinement from microED datasets than for single-crystal X-ray diffraction. The samples may also expand or degrade due to exposure to the electron beam. By concept, XRPD is the optimum method to arrive at the most precise unit-cell parameters (Chantler *et al.*, 2007 ▸), while microED and single-crystal X-ray diffraction are the methods preferred for fast indexing and the assignment of Bravais lattices.

A significant advantage of microED with respect to XRPD is that it can determine the positions of H atoms [see for example Yang *et al.* (2021 ▸)], leading to a complete experimental description of the hydrogen-bonding array, which is in general not obtainable from laboratory or synchrotron XRPD. Even neutron powder diffraction studies require deuteration (unless the amount of hydrogen is below a threshold level), due to the incoherent scattering cross section of hydrogen, which significantly increases the background intensity and may render the data useless.

For all three materials studied, all H atoms were directly located by microED from the Fourier difference maps (supporting information), and their positions were refined subject to a minimum number of restraints. This is of considerable interest (i) for cases in which tautomerism or chemical reactivity involving H atoms must be well understood, (ii) for the study of possible chemical mechanisms involving H atoms, (iii) for the structural description of hydrogen storage and hydrogen production materials (*e.g.* certain MOFs, hydrides), (iv) for materials with ferroelectric properties that are based on hydrogen-bonding arrays, (v) to distinguish pharmaceutical salts from cocrystals, *etc.*

**Figure 3:**
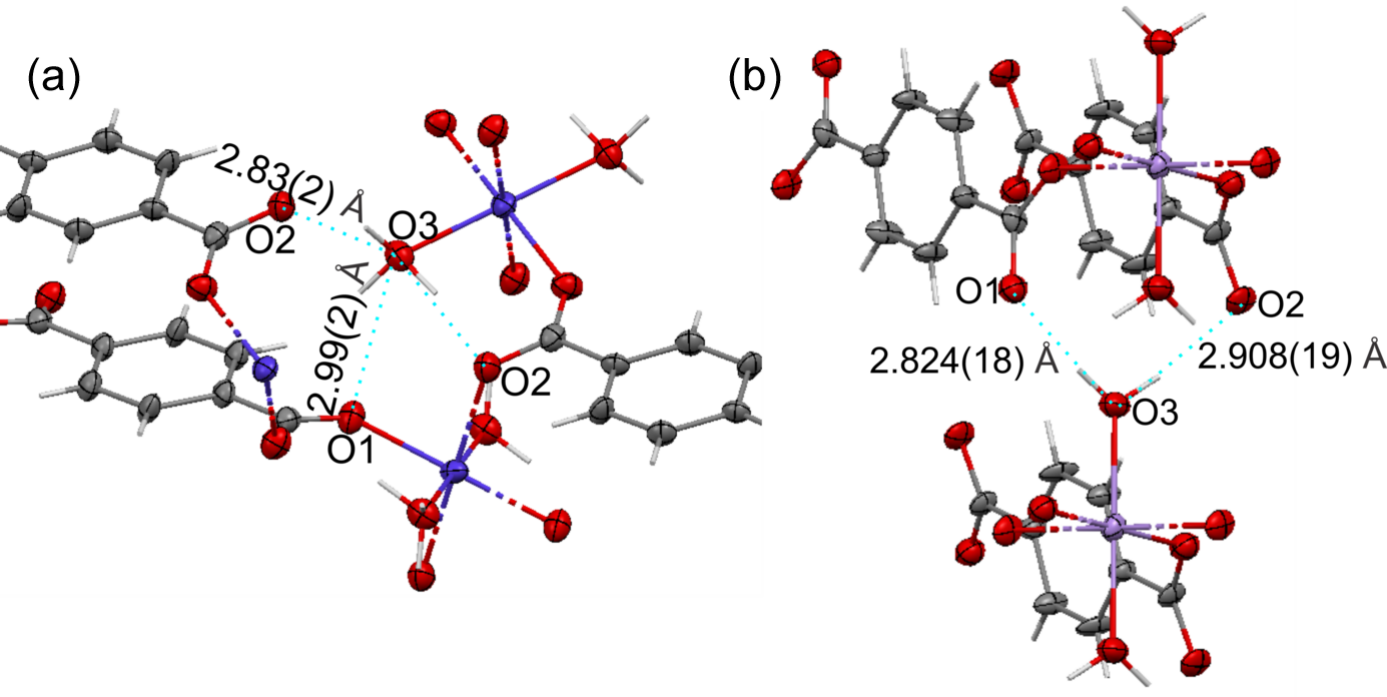
The hydrogen-bonding arrays in the crystal structures of (*a*) **II** and (*b*) **III**, as determined by microED (kinematic refinements). Hydrogen-bonding distances and angles are listed in Tables 4 ▸ and 5 ▸, respectively. Non-H atoms are represented as displacement ellipsoids at the 50% probability level and selected atom labels are shown. Hydrogen bonds are schematically represented with cyan dotted lines. C atoms are shown in grey, O in red, Co in blue, Mn in violet and H as light-grey sticks.

In the XRPD refinements of **II** and **III** (Kaduk, 2002 ▸), the H-atom positions were calculated by DFT using *CASTEP.* MicroED resulted in disordered H-atom positions for the water in **II**, but not in **III**. In **II** [Fig. 3 ▸(*a*)], the occupancy factors of the partially occupied positions H2*B* and H2*A* (both H atoms of O3_water_) are 0.88 (12) and 0.12 (12), respectively, while the H1 position (also bonded to O3_water_) is fully occupied. The experimentally determined hydrogen-bonding distances are shown in Fig. 3 ▸(*a*). Hydrogen bonding exists between O1_carboxylate_ and O3_water_—H1, as well as between O2_carboxylate_ and both O3_water_—H2*A* and O3_water_—H2*B*. These distances and the corresponding angles are compared in Table 4 ▸ with the values obtained by DFT. The experimental and calculated values are very close.

Note that the microED and DFT results do not agree for **III** since microED does not show H-atom disorder experimentally. This is represented in Fig. 3 ▸(*b*) and summarized in Table 5 ▸. It is possible that the analysis of the DFT results of **III** needs to be revised, since the third hydrogen-bond distance of 3.258 Å is unusually long; however, it is close to the distance between atoms O3 of two water molecules, 3.386 Å. Alternatively, a refinement of **III** at the dynamic electron diffraction theory level could be used to unambiguously rule out hydrogen disorder by microED.

Note that microED (at the kinematic level) leads to independently refined anisotropic atomic displacement parameters (ADPs) for all non-H atoms. XRPD typically affords only the refinement of isotropic ADPs (Lee & Xu, 2020 ▸). For organic materials, those are often (but not always) constrained to one or more group values. The ADPs of H atoms are refined using the riding model in both techniques. That is, they are subjected to constraints so that their values are 1.2 (or 1.5) times larger than the ADPs of the non-H atoms to which they are respectively attached.

For **II** and **III**, the isotropic ADPs calculated from XRPD for non-H atoms were constrained to four group values. These are the atoms of the phenyl ring, three carboxyl­ate atoms, the metal cation and the O atom of water. Their values ranged from 0.014 (3) to 0.026 (2) Å^2^ for **II** and from 0.012 (5) to 0.037 (5) Å^2^ for **III** (Kaduk, 2002 ▸). The corresponding values for **II** by microED (independently determined for each atom) were in the range 0.023 (2) to 0.031 (2) Å^2^. However, by microED (kinematic refinement) the heaviest atom (Co^2+^) has one of the lowest ADPs. The data of the kinematically refined structure were processed using *APEX4*, including an absorption correction. This generally improves the overall kinematic structure refinement but reduces the ADPs of the heavy atoms. Dynamically refined structures without absorption and extinction corrections are always preferred to retrieve more meaningful ADPs for the heavy elements. The ADP of Co^2+^ obtained by XRPD (the largest value for the non-H atoms of the structure) is not physically meaningful either, since the largest ADPs are expected for the lighter and more loosely bonded atoms (or groups of atoms) in the structure. In XRPD, the ADP values are, at least to some extent, correlated with the choice of background intensities and the X-ray absorption correction. They also depend on the counting statistics and the overall data quality at high angles, where the diffracted intensities contribute most to the ADPs.

For **III**, the microED values of the ADPs were found between 0.0223 (11) for Mn^II^ and 0.034 (2) for O3 (water), quite similar to those determined for **II**. Dynamic level refinements would improve the accuracy of these values. Like for **II**, the ADPs obtained by XRPD of the C and O atoms of **III** were lower than the ADP of Mn^II^, which is not physically meaningful, as indicated above.

### 3.3. Crystal structure of TAF-CNU-1 (I) by microED (refinement at the dynamic diffraction theory level)

The crystal structure of TAF-CNU-1 is now reported. Table 6 ▸ summarizes the crystallographic results. The structure was solved in the orthorhombic system with unit-cell parameters very similar to those fitting the laboratory XRPD data (Section 3.1). Synchrotron XRPD data were not collected. Fig. S4 shows a Le Bail fit of the XRPD data in the space group *Pbcn*, demonstrating that this phase composes the bulk of the powder synthesized. A few impurity peaks are due to an unidentified phase.

Table 7 ▸ compares the lattice parameters calculated by microED with those obtained by XRPD. Those are not equivalent within the estimated experimental error. As discussed for **II** and **III**, it is expected that the unit-cell parameters from microED will be less accurate than those from XRPD, for the same reasons previously outlined. We also compared the absolute values of the percent differences of the microED lattice parameters with the XRPD results, which were considered the true values for the calculations. These differences are small, between 1.1 and 2.1%, and comparable to the results for **II** and **III**.

An overlay of the laboratory XRPD pattern of TAF-CNU-1 with the calculated XRPD data using the crystal structure obtained from microED is shown in Fig. S7. This confirms that the experimental XRPD data had considerable preferred orientation effects, specifically in the first peak, indexed as 020. Preferred orientation is a factor increasing the difficulty of crystal structure determination from powders, or even the successful location of the nickel ions in the unit cell. This is important, because even if (after tedious work) the correct structural model could have been found, it would not have been possible to support it as an unambiguously determined crystal structure solution using only the experimental laboratory XRPD data. The use of reflection optics often renders laboratory XRPD data essentially untreatable for crystal structure determination in the presence of preferred orientation, for which typically synchrotron XRPD from transmission optics, using continuously spun capillaries in Debye–Scherrer geometry, is preferred instead. In such cases, preferred orientation effects are largely minimized (or avoided). High-quality powder diffraction intensities considerably facilitate obtaining a crystallographic model, refining it by the Rietveld method and reasonably supporting it without DFT calculations. Nevertheless, it must also be mentioned that the crystal structures of other MOFs of comparable complexity (Markun, 2022 ▸) have been solved and refined from synchrotron XRPD. The crystal structures of **II** and **III** were also refined with the Rietveld method (using preferred orientation corrections) from laboratory XRPD (Kaduk, 2002 ▸), after an initial model had been calculated *ab initio* for the isostructural compound containing Mg^II^ using synchrotron XRPD.

**Figure 4:**
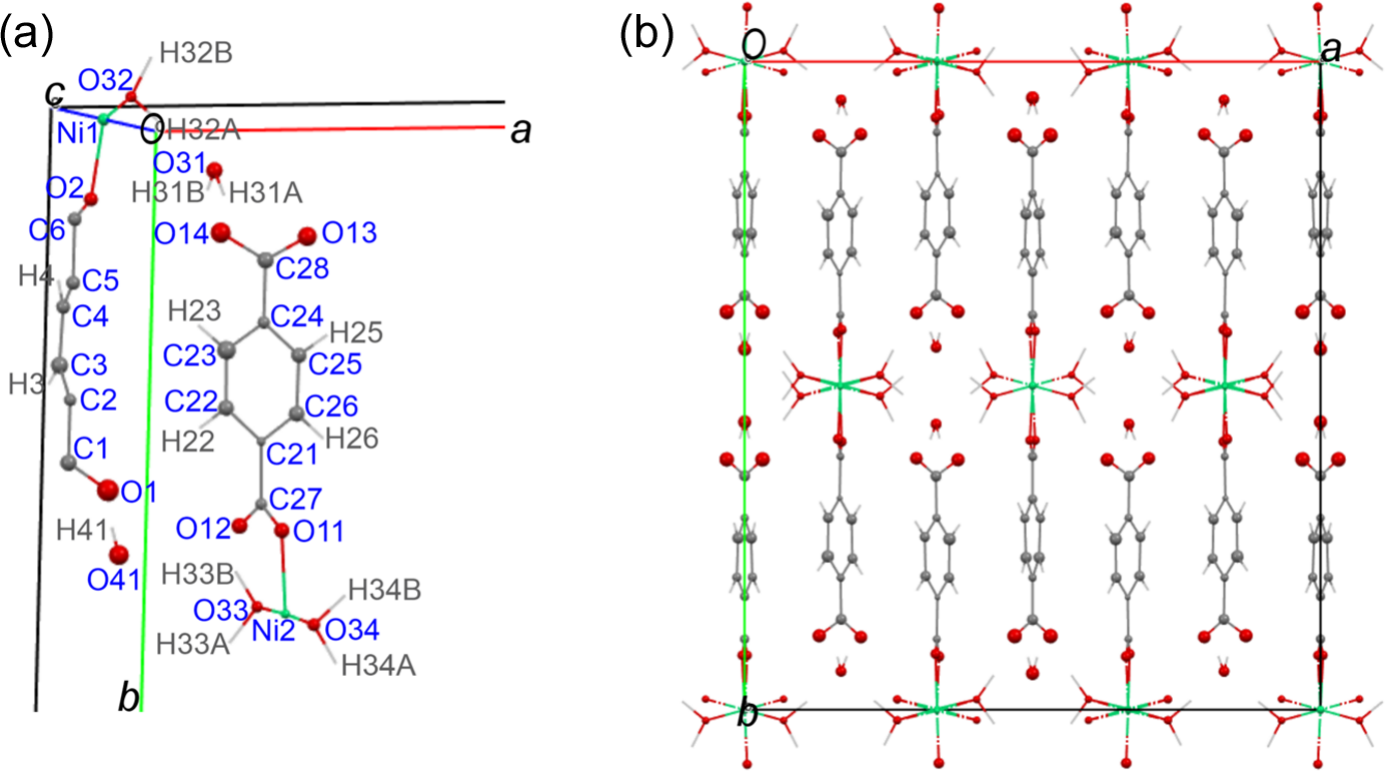
(*a*) The asymmetric unit of **I**, showing the atom labelling scheme and the crystallographic coordinate system. (*b*) The crystal structure of **I**, viewed along the *c*-axis direction. C atoms are shown in grey, O in red, Ni in green and H as light-grey sticks. Displacement ellipsoids of non-hydrogen atoms are represented at the 50% probability level.

The crystal structure of TAF-CNU-1 (**I**), including its asymmetric unit, is shown in Fig. 4 ▸. Charge balance in **I** implies that the tereph­thalate ions are fully deprotonated. Accordingly, the microED data did not show H atoms directly bonded to the carboxylate groups of the tereph­thalates in the Fourier difference map. The colour of the powder is pale green, pointing to an octahedral coordination for Ni^II^ (as experimentally observed), although Jahn–Teller distortions are also possible.

The Ni^II^ ions occupy two sets of atomic positions [Fig. 4 ▸(*a*)]: Ni1 is on an inversion centre at (0, 0, ½) with Wyckoff site 4*a* and Ni2 is on a general position of *Pbcn* with Wyckoff site 8*d*. This gives rise to a total of 12 Ni^II^ ions per unit cell. Carbon atoms C1, C2, C5 and C6 of a half-tereph­thalate fragment in the asymmetric unit [Fig. 4 ▸(*a*)] are located on special positions (0, *y*, ¼) with Wyckoff sites 4*c*. A twofold axis passing through these four atoms, together with C3, C4, O1 and O2 in general positions, generates the remaining four atoms of one tereph­thalate ion. These are two C atoms related by twofold symmetry to C3 and C4 and two O atoms analogously related to O1 and O2. The remaining crystallographically inequivalent full tereph­thalate ion does not have twofold symmetry along its longest molecular axis [Fig. 4 ▸(*a*)].

The relevant interatomic distances for the half-tereph­thalate ion of the asymmetric unit are 1.483 (10) Å for the C1—C2 single bond and 1.506 (10) Å for C5—C6 (also a single bond). The aromatic C C distances are shorter, 1.417 (7) Å × 2 and 1.383 (7) Å × 4. The respective values for the less symmetric (full) tereph­thalate anion are 1.469 (7) and 1.475 (6) Å for the C—C single bonds and 1.386 (7), 1.379 (7), 1.402 (7), 1.420 (7), 1.362 (7) and 1.440 (7) Å for the six shorter aromatic C C bonds.

**Figure 5:**
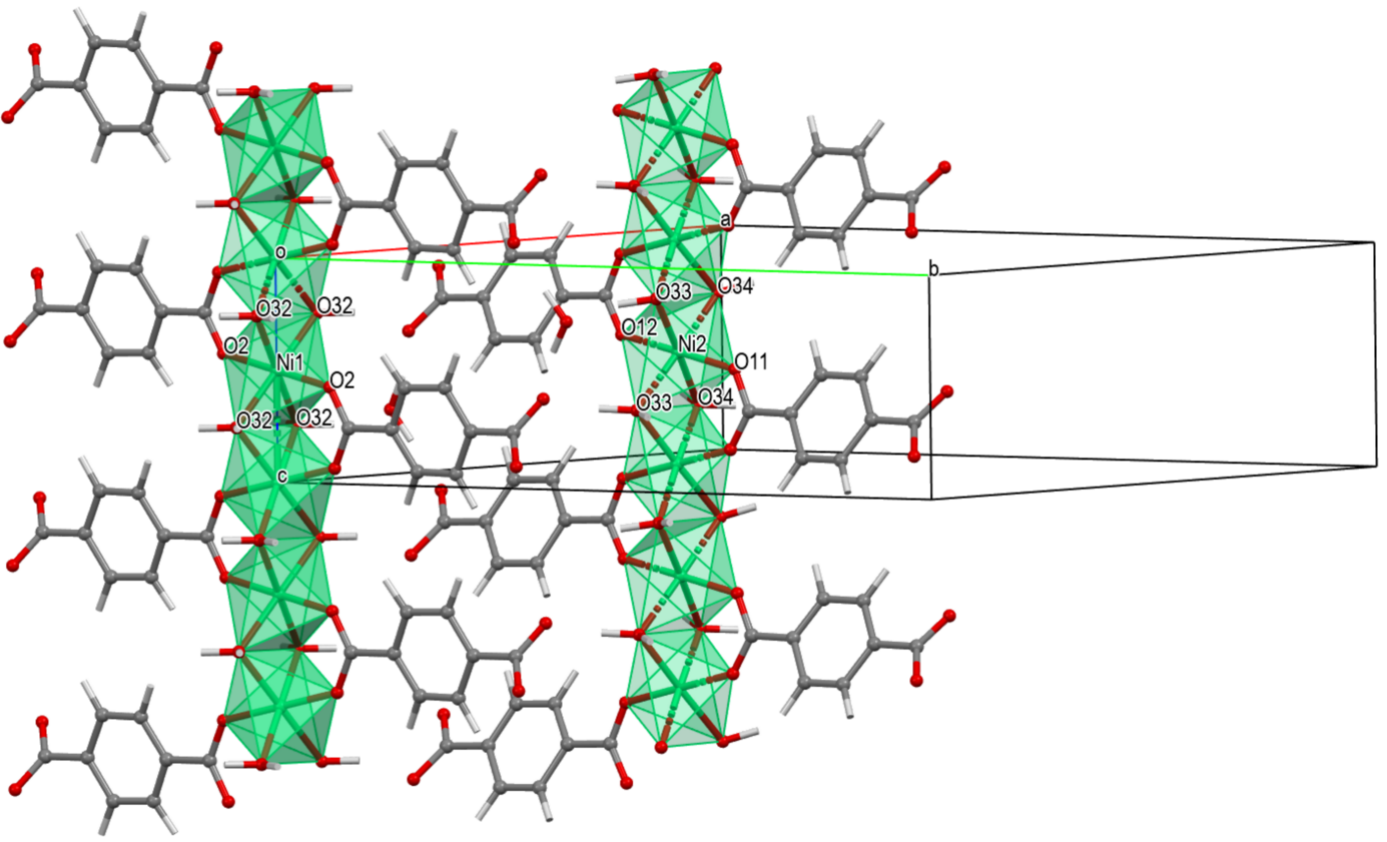
A view of the two crystallographically independent octahedron chains in TAF-CNU-1 (**I**), which run along the *c*-axis direction and are each derived from Ni1 or Ni2. The unit cell is also shown. Note that each octahedron chain and the coordinated terephthalates give rise to two crystallographically independent ribbon-type packing motifs of composition Ni(BDC)·2H_2_O, in which only one carboxyl­ate group of each terephthalate is part of the coordination spheres of Ni1 or Ni2, respectively. By crystal symmetry, the number of chains derived from Ni2 is twice that from Ni1.

Both nickel ions are at the centre of distorted octahedra with O atoms at all six vertices. This is shown in Fig. 5 ▸. Four O atoms are from water molecules and two from tereph­thalate anions. Differently than in **II** and **III**, the octahedra are not isolated in TAF-CNU-1 (**I**), but instead each octa­hedron shares two parallel edges with two adjacent octahedra in the same chain, giving rise to two sets of crystallographically independent chains, both running along the *c*-axis direction (Fig. 5 ▸). The Ni1 and Ni2 ions are coordinated to the respective crystallographically independent tereph­thalates (Fig. 4 ▸), forming two crystallographically independent packing motifs resembling a ‘ribbon’ or ‘tape’ (Fig. 5 ▸), also extending along the *c*-axis direction. By symmetry, Ni2 gives rise to twice the number of ribbon motifs (and octahedra) as Ni1.

Fig. 4 ▸(*b*) additionally shows that this crystal packing leads to hydro­phobic sections with organic molecules and π–π interactions, separated from rather hydro­philic regions with Ni^II^ centres and coordinating or hydrogen-bonding water mol­ecules. Since both crystallographically independent tereph­thalates only coordinate Ni^II^ through one of their carboxyl­ates, while the remaining carboxyl­ates are only hydrogen bonded to water, TAF-CNU-1 is deemed to be a pseudo-1D MOF. This segregated hydro­philic and hydro­phobic distribution of intermolecular interactions may be relevant for catalytic or other chemical reactivity properties of this new material. The corresponding anhydrous compound may also be an interesting candidate for ammonia or water capture applications, or as a simple desiccant.

The coordination spheres around both nickel centres are further described as follows. The two pairs of inversion-related Ni1—O32_water_ distances are 2.097 (6) and 2.124 (6) Å, while the two (inversion-related) Ni1—O2_carboxylate_ distances are shorter, 1.972 (6) Å. This agrees with an expected stronger interaction for Ni1—O2_carboxylate_ of greater ionic character (due to the negative charge on the carboxylate O atoms) than for Ni1—O32_water_. This bonding feature is also found in the edge-sharing octahedron chains involving Ni2. The pair of Ni2—O33_water_ bond lengths are 2.102 (7) and 2.146 (7) Å, while the pair of Ni2—O34_water_ bond distances are 2.122 (7) and 2.135 (7) Å. The shorter Ni2—O11_carboxylate_ and Ni2—O12_carboxylate_ bond distances are 1.985 (6) and 1.972 (6) Å, respectively, as expected from the charged nature of these centres. The Ni1—Ni1 distances are 3.113 Å, while Ni2—Ni2 are 3.114 Å, essentially equivalent. The pair of edges shared by adjacent octahedra in both crystallographically independent chains are the shortest among the 12 octahedron edges, 2.749 and 2.802 Å for Ni1 and Ni2, respectively.

**Figure 6:**
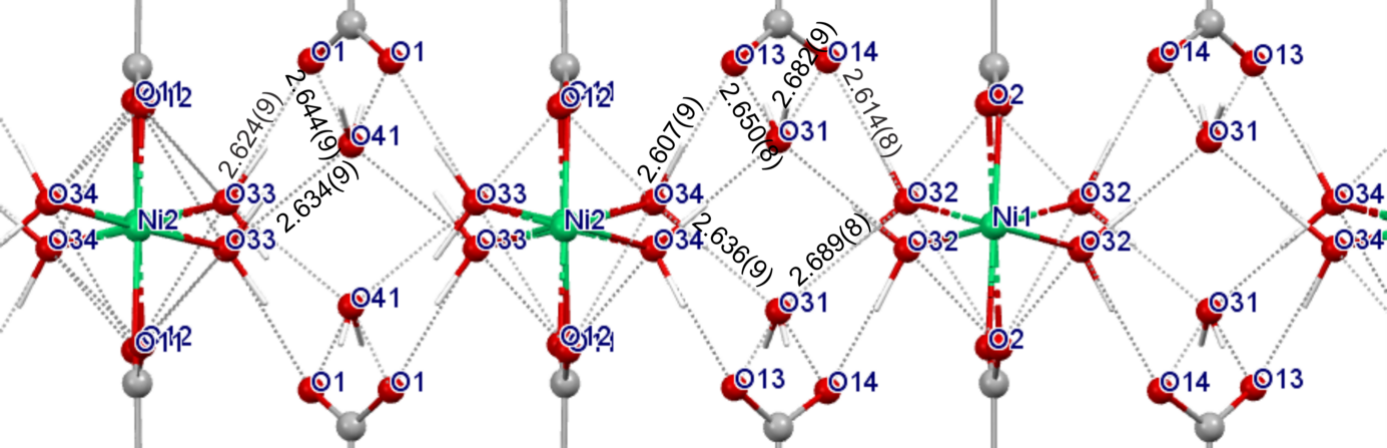
A view of the hydrogen-bonding array in TAF-CNU-1 (**I**) around the interstitial water molecules (O41_water_ and O31_water_), as calculated by *PLATON*. All hydrogen-bonding distances are in Å and are represented with dotted grey lines. All distances and angles are also listed in Table 8 ▸. Note that all carboxyl­ate O atoms are deprotonated and negatively charged. C atoms are shown in grey, O in red, Ni in green and H as light-grey sticks.

The hydrogen-bonding array is interesting as well and it is shown in Fig. 6 ▸. The parameters for all hydrogen bonds and associated H atoms are given in Table 8 ▸. Four water O atoms are on general positions (Wyckoff sites 8*d*) and a fifth water O atom, O41, is on a (0, *y*, ¼) special position (Wyckoff site 4*c*). Atoms O41_water_ and O31_water_ are not bonded to nickel, but rather fill interstitial spaces, hydrogen bonding to carboxylate O atoms and water molecules coordinated to nickel, so adding cohesion to the structure. These interstitial water molecules are fully ordered.

As a hydrogen-bond donor, O41_water_ is placed at 2.644 (9) Å from two O1_carboxylate_ atoms. Simultaneously, O41_water_ is the hydrogen-bond acceptor of two H33*A* atoms (from water molecules in the coordination spheres of Ni2 ions in adjacent chains) at 2.634 (9) Å each. This is shown in Fig. 6 ▸. Atom O31_water_ is the hydrogen-bond donor to atoms O13 and O14 at 2.650 (8) and 2.682 (9) Å, respectively, while it is the hydrogen-bond acceptor of atoms O34 and O32 at 2.636 (9) and 2.689 (8) Å, respectively.

Perhaps an unexpected feature of the hydrogen-bonding motif in TAF-CNU-1 is that the interstitial water molecules are hydrogen bonded directly to the negatively charged carb­oxy­lates of tereph­thalates, which coordinate to Ni^II^ through their remaining carboxylate groups. This structural feature has been observed in Cu(BDC)·3H_2_O as well (Cueto *et al.*, 1991 ▸). The crystal packing of TAF-CNU-1 is also very similar to that of Cu(BDC)·3H_2_O, but the *a* axis is tripled in TAF-CNU-1. A visual comparison of the two crystal structures with molecules coloured by symmetry is shown in Fig. S11. Both materials crystallize in the space group *Pbcn* and their *b* axes are around 22.9 Å, although the more symmetric Cu(BDC)·3H_2_O has only one set of crystallographically equivalent chains with a metal centre, a half-terephthalate fragment and a water molecule. This coordination environment, dictated by sym­metry, results in four molecules being coordinated to Cu^II^. The remaining crystallographically independent water occupies interstitial sites and contributes similarly to holding together adjacent Cu(BDC)·2H_2_O ‘ribbon’ motifs by hydrogen bonding.

### 3.4. TGA

The water content of TAF-CNU-1 was determined by TGA and the thermogram is shown in Fig. S12. The thermal decomposition of TAF-CNU-1 occurs in two steps, at around 400 and 420 °C. Mass loss due to dehydration also occurs in two steps, shown in detail in Fig. S13. The first event at around 149.8 °C leads to 17.51% mass loss, while the second event of 2.40% mass loss occurs at around 223 °C, resulting in a total mass loss of 19.9%. The calculated values for a monohydrate, a dihydrate and a trihydrate are 6.5%, 13% and 19%, respectively. Hence, the above measurements confirm that TAF-CNU-1 is a trihydrate, in agreement with the crystal structure determined by microED.

The thermograms of **II** and **III** are shown in the supporting information (Figs. S14 and S15, respectively). Both materials are dihydrates, and their water contents agree with the values determined by microED and XRPD (Kaduk, 2002 ▸). Compound **II** showed three mass losses at 120, 240 and 400 °C. The water content of **II** was approximately 11.4% by weight. The first weight loss of **III** was observed approximately in the 140–180 °C temperature range. Compound **III** contained approximately 13.2% of water by weight.

### 3.5. FT-IR

**Figure 7:**
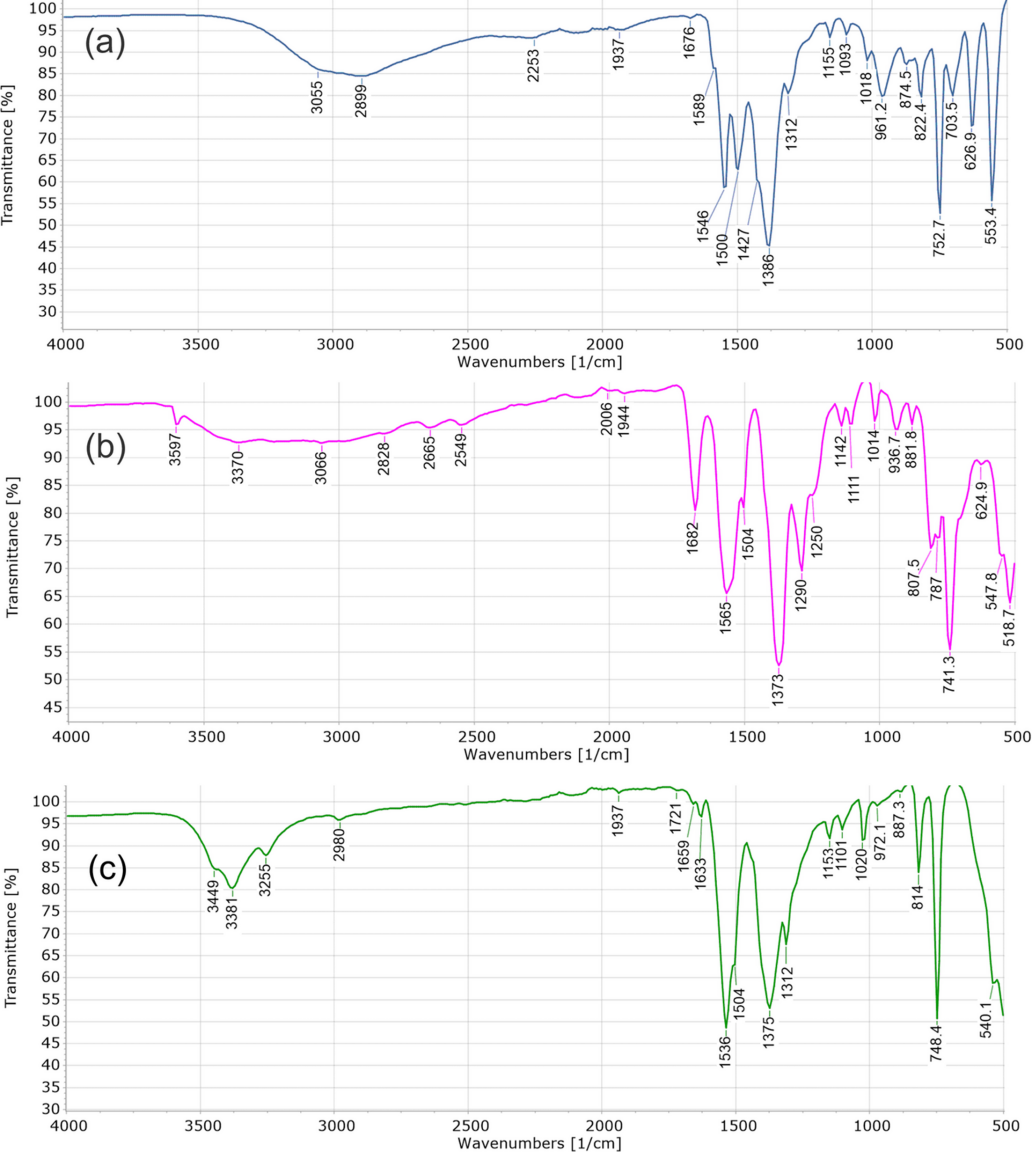
FT-IR spectra of (*a*) TAF-CNU-1 (**I**), (*b*) **II** and (*c*) **III**. The wavenumbers of selected bands (in cm^−1^) are shown.

The FT-IR spectra of TAF-CNU-1 (**I**), **II** and **III** are shown in Fig. 7 ▸. All three materials have a very broad and moderately intense absorption band in the 2500–3500 cm^−1^ interval, due to the stretching vibrations of hydrogen-bonded O—H groups in water. This band is centred at lower wavenumbers for TAF-CNU-1 than for **II** and **III**. This can be tentatively explained by the different water coordination in TAF-CNU-1, which contains interstitial water not directly bonded to the metal centres, so the corresponding absorptions occur at lower energies. Note also the differences when comparing only the bands of **II** and **III**. An overall broader band is observed in **II**, which contains disordered H atoms in its water molecules, and a narrower absorption is seen in **III**, without H-atom disorder. Other absorptions also appear in this wavenumber interval, such as weak bands due to C—H stretching at around 3000 cm^−1^.

In the 1000–2000 cm^−1^ wavenumber interval, wherein the absorptions of double and single bonds (without hydrogen) typically occur, the absence of a very strong (or the strongest) band corresponding to a carbonyl stretching (typically at around 1700 cm^−1^) is noticeable. This points to the presence of carboxylate groups in doubly deprotonated tereph­thalate ions, as determined by microED.

All three materials showed strong bands in the 1000–2000 cm^−1^ interval, although it is challenging to assign individual bond vibrations to particular bands, since infrared active vibrations tend to be coupled in this region. Such coupling has been reported for tereph­thalic acid, supported by *ab initio* DFT calculations (Téllez *et al.*, 2001 ▸). Nonetheless, the spectra of **II** and **III** resemble each other in this wavenumber interval, as one could intuitively expect from isostructural compounds. For **III**, the two strongest bands are at 1536 and 1375 cm^−1^, with weak shoulders at 1504 and 1312 cm^−1^, respectively. These strong bands have analogues in **II**, observed at 1565 and 1373 cm^−1^, respectively, with a corresponding weaker shoulder at 1504 cm^−1^ and another band at 1290 cm^−1^. In **II**, there is an additional strong absorption at 1682 cm^−1^. It is interesting to compare these spectral features with those of other similar MOFs, since the medium to strong absorptions of the carboxylate groups and *M*—O groups (*M* = Ni, Co or Mn) have not yet been assigned. The asymmetric stretching of carboxylate groups in tere­phthalates has been assigned to moderately strong bands at 1686 cm^−1^ (Tanak *et al.*, 2013 ▸), while the symmetric stretching vibrations have been assigned to bands at 1372 cm^−1^, corresponding to the strong bands indicated above at 1375 and 1373 cm^−1^ for **III** and **II**, respectively, and the strong absorption at 1386 cm^−1^ for TAF-CNU-1 (**I**). As in the work of Tanak *et al.* (2013 ▸), and also supported by DFT calculations, moderately strong bands at around 745 cm^−1^ can be tentatively assigned to scissoring vibrations of the carboxylates. Those show at 753, 741 and 748 cm^−1^ for **I**, **II** and **III**, respectively. The reports by Tanak *et al.* (2013 ▸) and Téllez *et al.* (2001 ▸) do not mention bands at around 1546, 1565 and 1536 cm^−1^ (for **I**, **II** and **III**, respectively) possibly ascribed to tereph­thalates, although a band at 1560 cm^−1^ has been assigned to the carboxylates of Fe–tereph­thalate MOFs, wherein Fe has been partially substituted by Mn, Co or Ni (Sun *et al.*, 2017 ▸). That study also mentions that the bands assigned to *M*—O vibrations (*M* = Mn, Fe, Co or Ni) appear in the 509–544 cm^−1^ interval.

### 3.6. SEM analysis

**Figure 8:**
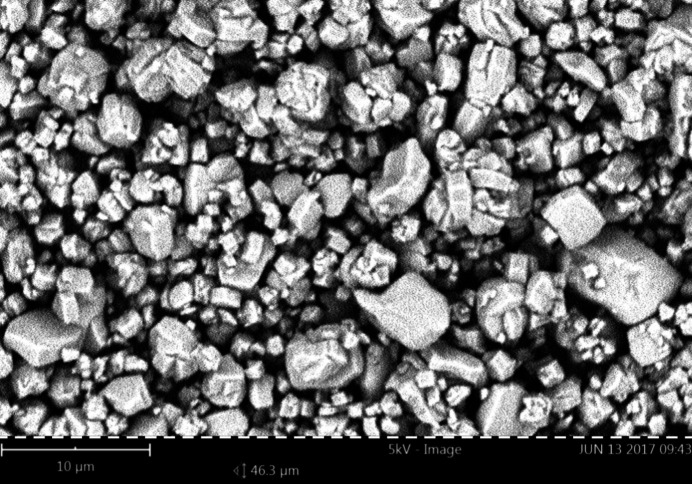
SEM image of TAF-CNU-1 (**I**).

The three materials show well crystallized powders by SEM, in agreement with all the diffraction experiments (by XRPD and microED). Fig. 8 ▸ shows an SEM image of TAF-CNU-1 (**I**), while Fig. S16 shows the corresponding images for **II** and **III**. The particle morphologies are of the block type in all cases. The particle sizes of TAF-CNU-1 are in the 8–10 µm range (for the largest particles) down to 1 µm (or less) for the smallest particles.

## 4. Conclusions

This work reports the crystal structure of a new material, Ni(C_8_H_4_O_4_)·3H_2_O, a pseudo-1D MOF. This material was synthesized in aqueous media under ambient conditions, leading to sustainable ‘green’ chemistry and the preparation of substances that are stable under atmospheric moisture content. Its anhydrous counterpart may find applications in ammonia or water uptake processes.

The crystal structure of Ni(C_8_H_4_O_4_)·3H_2_O could not be solved from laboratory XRPD data due to the presence of impurity peaks and preferred orientation effects, but it was solved and refined (at the dynamic electron diffraction theory level) from microED measurements from micrometre-sized crystallites in the powder. The redetermination of the crystal structures of *M*(C_8_H_4_O_4_)·2H_2_O (*M* = Co^II^, Mn^II^) by microED (refined at the kinematic level) allowed their comparison with published XRPD results supported by DFT calculations. It is concluded that crystal structure determination by microED has a vast potential for use. It becomes an efficient high-value tool when only very small amounts of powders are available for study or when the application of traditional X-ray diffraction methods leads to ambiguities requiring additional data for resolution, for example the determination of hydrogen-bonding motifs using XRPD.

XRPD and microED complement each other very well for crystal structure analysis. The former can lead straightforwardly to the composition of the bulk powder (by qualitative and quantitative phase analysis), lattice parameters of unrivalled accuracy and microstructural properties determined from peak profile shapes and peak broadening. MicroED shines at rendering high-quality crystal structures and atomic positions independently determined from a three-dimensional set of diffracted intensities (as in single-crystal X-ray diffraction), including H-atom positions directly refined from electron-density maps, occupancy factors and anisotropic atomic displacement parameters for the non-hydrogen atoms. Absolute stereochemistry determinations are also possible using microED.

It is undoubtedly advantageous that microED experiments dramatically shift the limiting restriction of minimum crystal size for single-crystal X-ray diffraction. They can be carried out even on nanomaterials and on samples made of very small quantities of powders. Disadvantages of microED exist, such as lower-quality values of the unit-cell parameters, the need of dynamic refinements to reduce the residuals (which may require time-consuming non-routine calculations) and the use of high vacuum for data collection, which may constrain the measurement conditions to cryogenic temperatures to stabilize certain materials. At present, the knowledge of routine experimental corrections and other commonly observed effects in microED data is less developed than that for X-ray diffraction techniques. Nevertheless, dedicated devices for microED are a significant step forward in resolving experimental drawbacks and increasing microED’s uses. This work and several other published reports point to a bright future for the routine application of microED for crystal structure determination of materials available at the micrometre and nanometre size scales. In the future, this may even be routinely achieved in the chemical laboratory (academic, forensic, pharmaceutical, natural products *etc.*).

## 5. Related literature

The following references are cited only in the supporting information: Bruker (2016 ▸); Bruker (2019 ▸); Doyle & Turner (1968 ▸); Huang *et al.* (2021 ▸). 

## Supplementary Material

Crystal structure: contains datablock(s) global, I, II, III. DOI: 10.1107/S1600576724012068/te5145sup1.cif

Additional experimental details and supplementary figures. DOI: 10.1107/S1600576724012068/te5145sup2.pdf

CCDC references: 2409538, 2415838, 2415839

## Figures and Tables

**Table 1:** Crystallographic data for **II** and **III** obtained by microED at room temperature, implementing least-squares refinements at the kinematic electron diffraction theory level

Chemical formula	Co(C_8_H_4_O_4_)·2H_2_O	Mn(C_8_H_4_O_4_)·2H_2_O
*M*_r_	259.07	255.08
Crystal system	Monoclinic	Monoclinic
Space group symmetry	*C*2/*c*	*C*2/*c*
Space group number	No. 15	No. 15
*a* (Å)	18.22 (11)	18.71 (8)
*b* (Å)	6.54 (4)	6.57 (3)
*c* (Å)	7.28 (4)	7.37 (3)
α (°)	90.0	90.0
β (°)	98.867 (15)	99.270 (13)
γ (°)	90.0	90.0
*V* (Å^3^)	857 (9)	894 (7)
*Z*	4	4
λ (Å)	0.02851	0.02851
*T* (K)	293	293
Pressure (kPa)	4 × 10^−7^	4 × 10^−7^
*R* (%)	20.44 (839 reflections)	18.17 (893 reflections)
w*R*_2_ (%)	52.42 (1096 reflections)	46.08 (1049 reflections)
Main shell† and overall‡ completeness (%)	90.8 and 81.7	85.2 and 75.2

† Main shell resolution range up to 0.86 Å.

‡ Overall resolution up to 0.7 Å.

**Table 2:** A comparison of *M*—O distances within octahedra (*M* = Co^II^ or Mn^II^ for **II** or **III**, respectively) solved from microED (kinematic diffraction approach) with those calculated using *PLATON* from the published CIF files by XRPD and those from DFT calculations (Kaduk, 2002 ▸)

Material	*M*—O (Å) by microED	*M*—O (Å) by XRPD	*M*—O (Å) by DFT
**II**	2.149 (16) × 2	2.120 (12) × 2	2.308 × 2
	2.093 (16) × 2 (water)	2.106 (10) × 2 (water)	2.012 × 2
	2.078 (16) × 2	2.054 (12) × 2	1.981 × 2

**III**	2.195 (14) × 2 (water)	2.197 (5) × 2	2.107 × 2
	2.180 (14) × 2	2.195 (5) × 2 (water)	2.065 × 2
	2.148 (13) × 2	2.189 (6) × 2	1.997 × 2

**Table 3:** The monoclinic unit-cell parameters *a*, *b*, *c* and γ (in Å and °) and unit-cell volume *V* (in Å^3^) for **II** and **III**, determined by XRPD and microED (kinematic diffraction model)

Material	*a*, *b*, *c*, γ and *V* by microED	*a*, *b*, *c*, γ and *V* by XRPD†	*a*, *b*, *c*, γ and *V* by XRPD‡
**II**	18.22 (11)	18.2731 (8)	18.269 (1)
	6.54 (4)	6.5417 (3)	6.5424 (3)
	7.28 (4)	7.2966 (3)	7.2948 (3)
	98.867 (15)	98.653 (2)	98.653 (3)
	857 (9)	862.29 (7)	861.95 (8)

**III**	18.71 (8)	18.7213 (13)	18.688 (4)
	6.57 (3)	6.5960 (4)	6.601 (1)
	7.37 (3)	7.4035 (6)	7.400 (1)
	99.270 (13)	99.287 (3)	99.223 (5)
	894 (7)	902.24 (11)	901.0 (3)

† Kaduk (2002 ▸).

‡ This work. Le Bail fits and their agreement factors are shown in the supporting information.

**Table 4:** O3_water_—H⋯O_carboxylate_ hydrogen-bond distances (Å) and the respective angles (°) for **II**, by microED (kinematic refinement) and by DFT using *CASTEP*

O3_water_—H⋯O_carboxylate_	MicroED (Å, °)	DFT† (Å, °)
O3—H2*B*⋯O2	2.83 (2), 159 (5)	2.834, 149.6
O3—H1⋯O1	2.99 (2), 128 (4)	2.986, 131.5
O3—H2*A*⋯O2	2.924‡, 163.38‡	2.871, 165.4

† Kaduk (2002 ▸).

‡ Standard uncertainties not reported by *PLATON*.

**Table 5:** O3_water_—H⋯O_carboxylate_ hydrogen-bond distances (Å) and the respective angles (°) for **III**, by microED (kinematic refinement) and by DFT using *CASTEP*

O3_water_—H⋯O_carboxylate_	MicroED (Å, °)	DFT† (Å, °)
O3—H1⋯O1	2.824 (18), 173 (8)	2.918, 166.0
O3—H1*A*⋯O2	2.908 (19), 165 (6)	2.963, 145.4
O3—H1⋯O	–	3.258, 135.9

† Kaduk (2002 ▸).

**Table 6:** Crystallographic results for TAF-CNU-1 (**I**) from microED (refinement at the dynamic diffraction theory level)

Chemical formula	Ni(C_8_H_4_O_4_)·3H_2_O
*M*_r_	276.85
Crystal system	Orthorhombic
Space group symmetry	*Pbcn*
Space group number	No. 60
*a* (Å)	20.31 (5)
*b* (Å)	22.85 (5)
*c* (Å)	6.226 (14)
α (°)	90.0
β (°)	90.0
γ (°)	90.0
*V* (Å^3^)	2889 (12)
*Z*	12
λ (Å)	0.02851
*T* (K)	293
Pressure (kPa)	4 × 10^−7^
*R* (%)	11.57 (2038 reflections)
w*R*_2_ (%)	24.67 (6918 reflections)
*S*	1.746
No. of parameters	391
Main shell† and overall‡ completeness (%)	97.8 and 76.8

† Main shell resolution range up to 0.86 Å.

‡ Overall resolution up to 0.7 Å.

**Table 7:** *a*, *b* and *c* unit-cell parameters (α, β and γ = 90.0°) and unit-cell volume (*V*) of TAF-CNU-1 (**I**) determined by microED and laboratory XRPD, at room temperature in both cases

Lattice parameter	MicroED (dynamic refinement)	X-ray powder diffraction
*a* (Å)	20.31 (5)	20.737 (5)
*b* (Å)	22.85 (5)	23.318 (5)
*c* (Å)	6.226 (14)	6.2983 (15)
*V* (Å^3^)	2889 (12)	3045.4 (13)

**Table 8:** Hydrogen-bonding distances (Å) and angles (°) for TAF-CNU-1 (**I**), as determined by *PLATON*

O_donor_—H⋯O_acceptor_	O_donor_—H (Å)	H⋯O_acceptor_ (Å)	O_donor_⋯O_acceptor_ (Å)	O_donor_—H⋯O_acceptor_ (°)
O31—H31*A*⋯O14	1.051 (15)	1.656 (17)	2.682 (9)	164 (2)
O31—H31*B*⋯O13	1.039 (16)	1.619 (16)	2.650 (8)	171.1 (17)
O32—H32*A*⋯O31	1.053 (16)	1.655 (16)	2.689 (8)	166.0 (17)
O32—H32*B*⋯O14	1.055 (13)	1.600 (15)	2.614 (8)	159.3 (17)
O33—H33*A*⋯O41	1.077 (13)	1.617 (15)	2.634 (9)	155.2 (12)
O33—H33*B*⋯O1	1.046 (13)	1.588 (14)	2.624 (9)	170.3 (15)
O34—H34*A*⋯O13	1.045 (16)	1.567 (16)	2.607 (9)	172.7 (16)
O34—H34*B*⋯O31	1.062 (17)	1.577 (17)	2.636 (9)	174.1 (17)
O41—H41⋯O1	1.03 (2)	1.62 (2)	2.644 (9)	170.1 (19)
