# Supplementary material for: A comparison of microcrystal electron diffraction and X-ray powder diffraction for the structural analysis of metal–organic frameworks
Source: J Appl Crystallogr. 2025 Feb 28;58(Pt 2):398–411. doi: 10.1107/S1600576724012068 (PMC11957409; doi:10.1107/S1600576724012068)
Supplement: Supplementary file 2 [file j-58-00398-sup2.pdf]

JOURNAL OF
APPLIED
CRYSTALLOGRAPHY

Volume 58 (2025)

Supporting information for article:

A comparison of microcrystal electron diffraction and X-ray powder diffraction for the structural analysis of metal-organic frameworks

Erik Biehler, Silvina Pagola, Daniel Stam, Johannes Merkelbach, Christian Jandl and Tarek M. Abdel-Fattah

S1. Laboratory X-ray powder diffraction

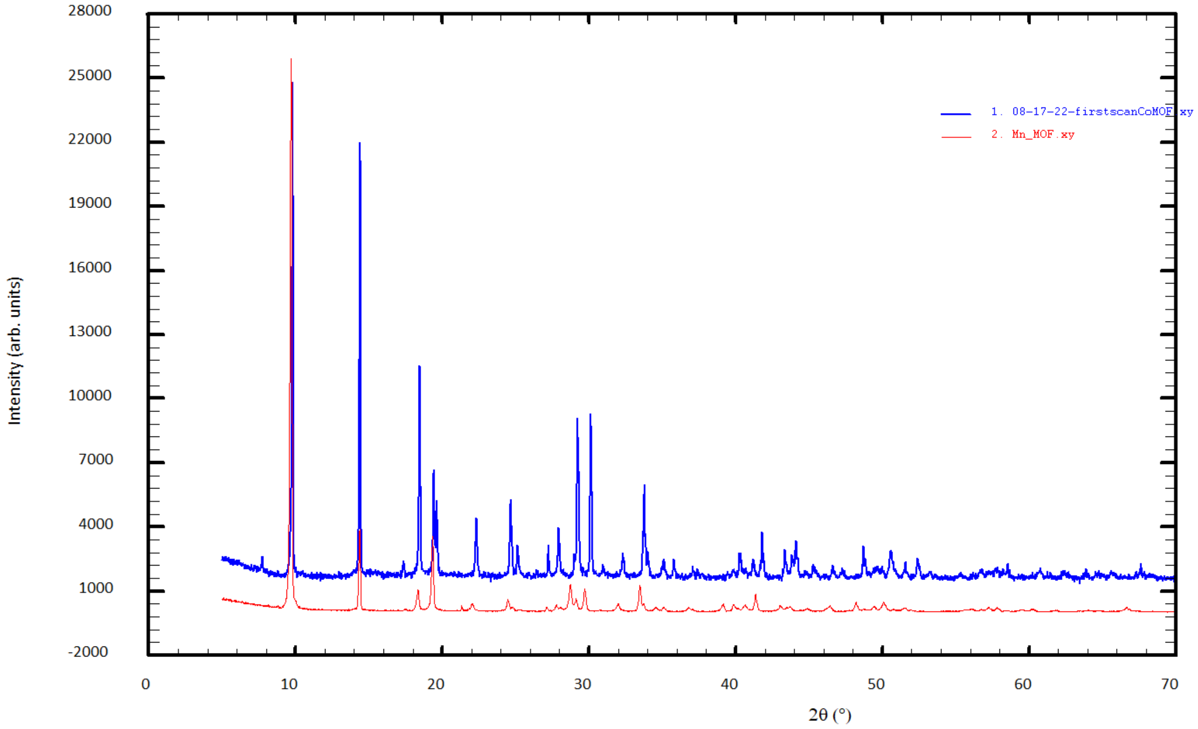

Figure S1 Overlay of the XRPD data of **II** (blue line) and **III** (red line). The scale of the pattern of **II** was multiplied by three for comparison purposes only. Note that although some weak peaks of **II** do not have a match in the data of **III**, the overall aspect of the positions and relative intensities suggests the possibility of **II** and **III** being isostructural.

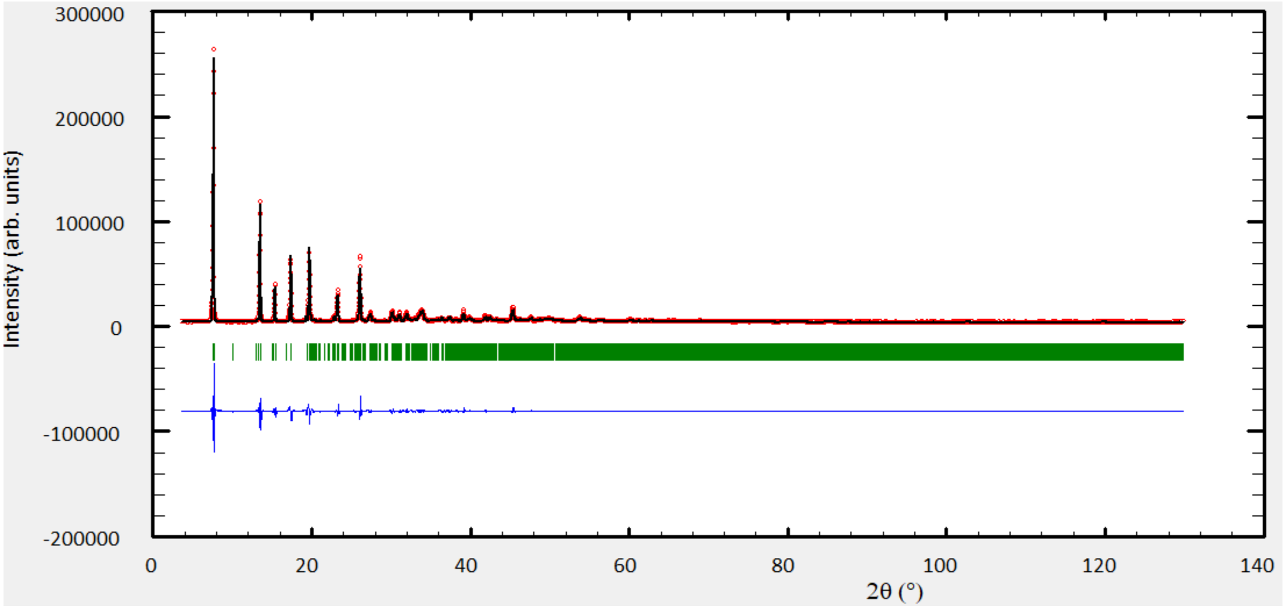

Figure S2 Le Bail fit of the XRPD pattern of TAF-CNU-1 (**I**) using the monoclinic unit cell a = 13.6567 Å, b = 22.9964 Å, c = 4.5895 Å, α = 90.00°, β = 92.4557°, γ = 90.00°, and V= 1430 $Å^3$, with indexing figure of merit 9 and the space group $P2_1/a$. The experimental data are shown with red circles, the calculated profile is shown with a black line, and the difference between observed and calculated data is shown in blue at the bottom. The vertical green symbols indicate allowed Bragg peak positions. The agreement factors were R_{wp} = 22 % and χ^2 = 42.

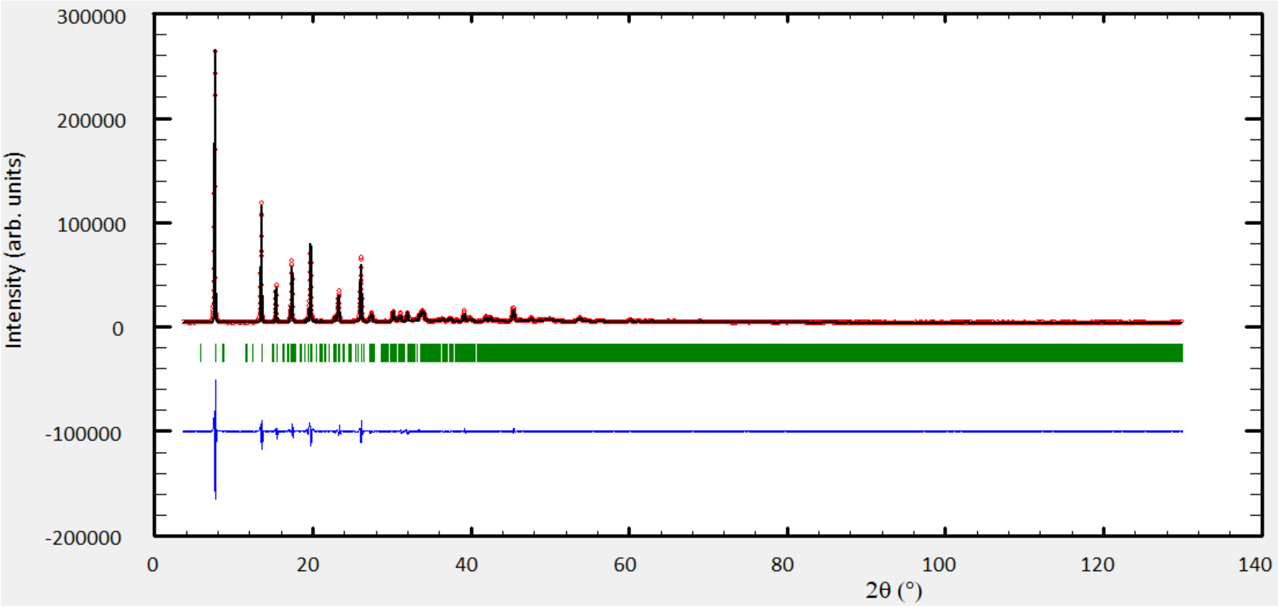

Figure S3 Le Bail fit of the XRPD pattern of TAF-CNU-1 (**I**) using the orthorhombic unit cell a = 20.451 Å, b = 22.9816 Å, c = 6.1889 Å, α = 90.00°, β = 90.00°, γ = 90.00°, M = 8 and V = 2897 Å^3 in the space group *Pbnn*. The experimental data are shown with red circles, the calculated profile is shown with a black line, and the difference between observed and calculated data is shown in blue at the bottom. The vertical green symbols indicate allowed Bragg peak positions. The agreement factors were R_{wp}= 26.2 % and χ^2= 59.

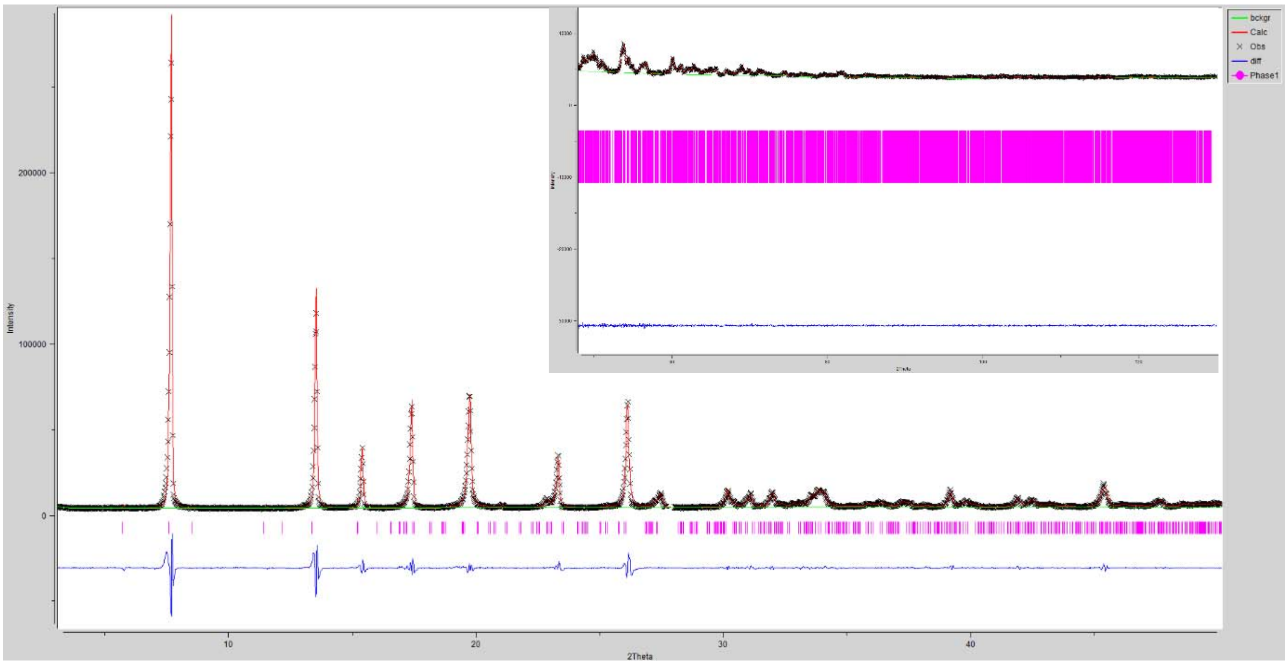

Figure S4 Le Bail fit of the XRPD pattern of TAF-CNU-1 (**I**) using the orthorhombic unit cell indicated in Section 3.1 in the space group *Pbcn*. The experimental data are shown with x marks, the calculated profile is shown with a red line, and the difference between observed and calculated data is shown in blue at the bottom. The vertical magenta symbols indicate allowed positions for Bragg peaks. The green line represents the background intensities. The inset shows an enlargement of the high-angle region. The agreement factors were R_{wp} = 6.30 % and χ^2 = 22.05.

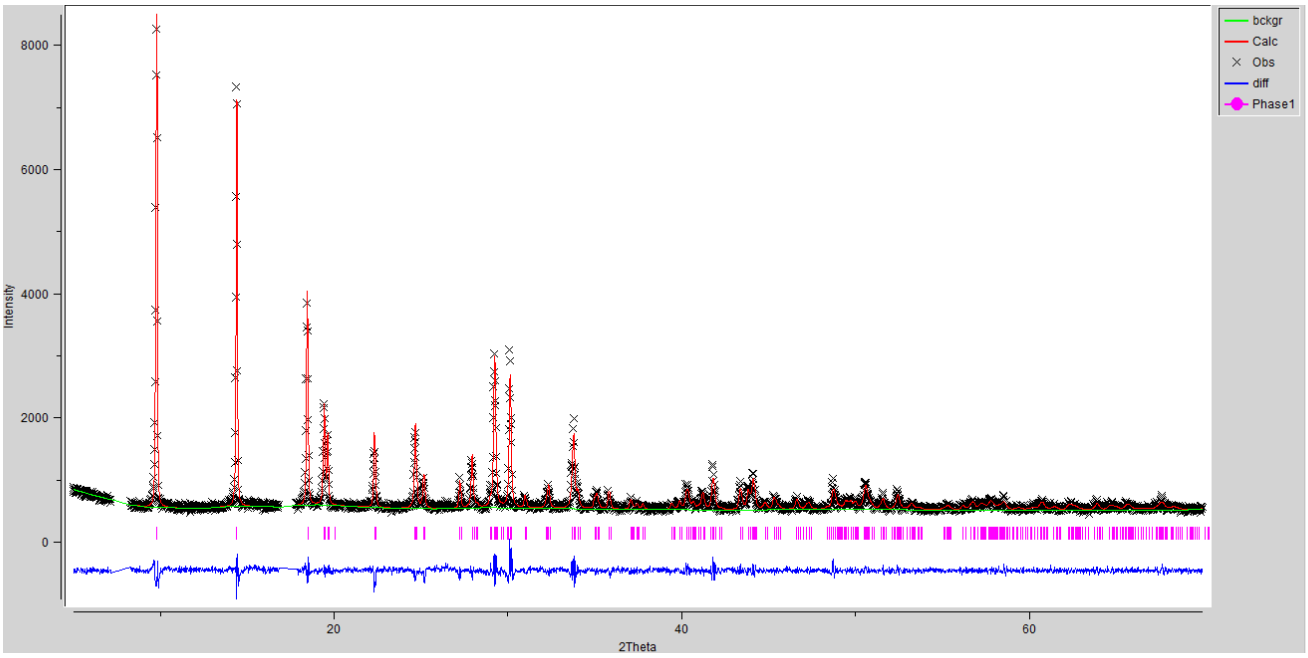

Figure S5 Le Bail fit of the XRPD data of **II** to confirm the validity of the unit-cell parameters. Two angular regions were excluded due to the presence of impurities. The experimental data are shown with black x symbols, the calculated profile is shown with a red line, and the difference between observed and calculated data is shown in blue at the bottom. The vertical magenta symbols indicate allowed Bragg peak positions. The green line represents the background intensities. R_{wp} = 5.75 %, χ^2 = 2.199. Note that refining the unit-cell parameters (initially from microED) only after the rest of the fit was essentially finished led to shifted peaks and χ^2 = 3.107.

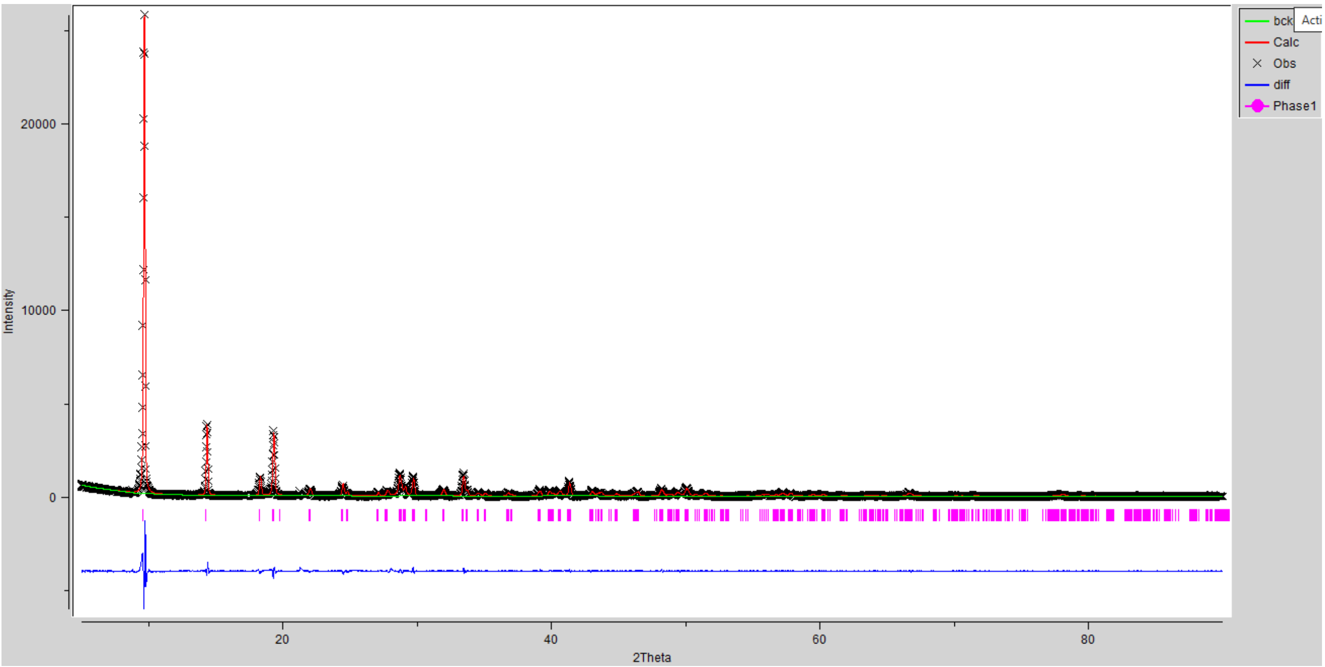

Figure S6 Le Bail fit of the XRPD data of **III** to confirm the validity of the unit-cell parameters. Two small angular regions were excluded due to the presence of impurities. The experimental data are shown with black x symbols, the calculated profile is shown with a red line, and the difference between observed and calculated data is shown in blue at the bottom. The vertical magenta symbols indicate allowed Bragg peak positions. The green line represents the background intensities. R_{wp} = 12.13 %, χ^2 = 2.880.

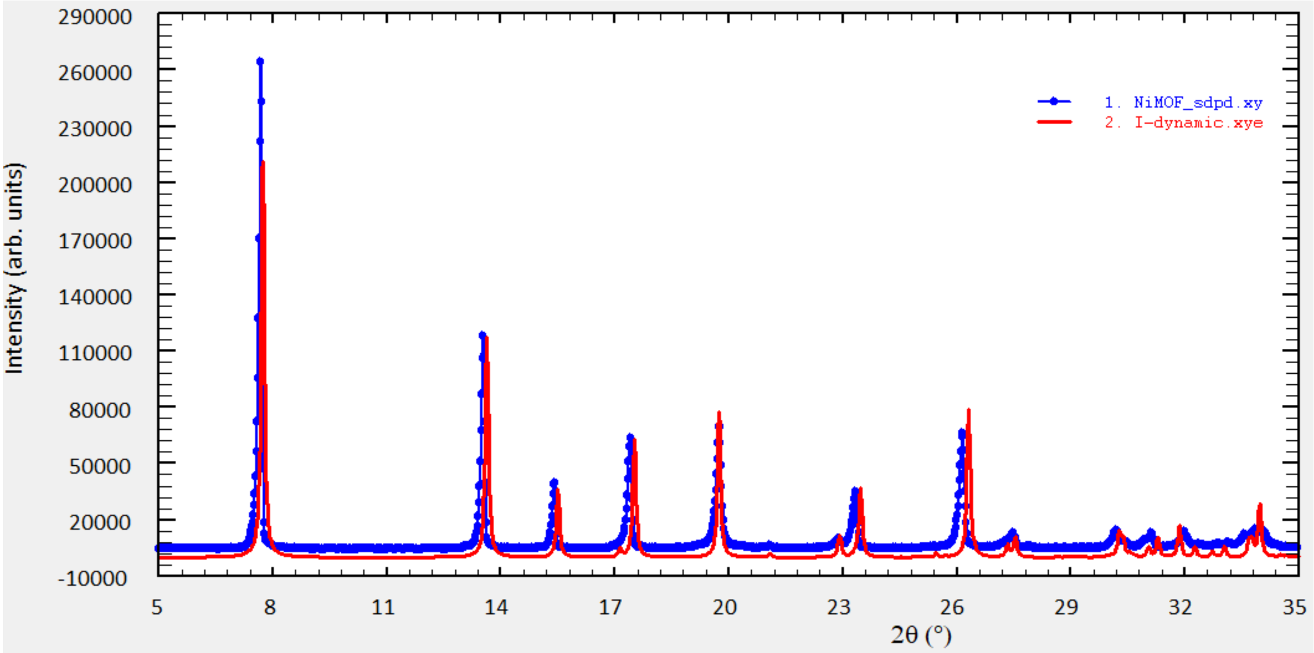

Figure S7 Overlay of the low-angle region of the XRPD pattern of TAF-CNU-1 (blue line with circles) and the calculated XRPD pattern from the microED results (red line). Note the difference in relative intensities of the first peak, (020), showing preferred orientation in the XRPD data.

S2. Micro-crystal electron diffraction (microED) additional experimental details

The data for the kinematically refined structure solution were processed and evaluated using the *APEX4* software (Bruker 2022). After unit-cell determination, the frames were integrated and corrected for Lorentz effects, scan speed, background and beam absorption using *SAINT* (Bruker 2019) and *SADABS* (Bruker 2016). Space group determination was based on the systematic absences, *E* statistics and successful structural refinements. The crystal structure was solved using *ShelXD* and refined with *ShelXL* (Sheldrick, 2015) in conjunction with *ShelXle* (Hübschle *et al*., 2011).

Least-squares refinements were carried out within the kinematic approximation by minimizing $\Sigma w(F_{obs}^2-F_{calc}^2)^2$ with the *ShelXL* weighting scheme using neutral electron scattering factors (Doyle *et al.*, 1968). Non-H atoms were refined with anisotropic displacement parameters. Rigid-body restraints were employed to ensure convergence within physically reasonable limits. H atoms (except on heteroatoms) were placed in calculated positions and refined with a riding model based on neutron diffraction distances and $U_{iso}(H) = 1.2 \cdot U_{eq}(C)$. For hydroxyl H atoms, neutron distances and $U_{iso}(H) = 1.5 \cdot U_{eq}(O)$ were used, and the angles were freely refined.

The data for the dynamic refinement (performed after convergence of the kinematic refinement) were processed using the *PETS2* software package (Palatinus *et al.*, 2019). After unit-cell determination the frames were integrated, corrected for experimental parameters as in the kinematic fits and merged into overlapping virtual frames (Huang *et al.,* 2021). The dynamic refinement was performed using *JANA2020* (Petříček *et al*., 2014) starting with the structure obtained from the kinematic refinement as the initial model. Least-squares refinements were carried out by minimizing $\Sigma w(I_{obs}-I_{calc})^2$, based on dynamic diffraction intensities and assuming uniform thickness of the crystals. All atoms were refined with isotropic displacement parameters. H atoms (except on heteroatoms) were placed in calculated positions and refined with a riding model based on neutron diffraction distances and $U_{iso}(H) = 1.2 \cdot U_{eq}(C)$. For hydroxyl H atoms, neutron distances and $U_{iso}(H) = 1.2 \cdot U_{eq}(O)$ were used, and the angles were freely refined.

The unit-cell parameters reported were taken from the best data set with the lowest R_{int}. Usually, five to ten data sets are collected per sample, and two to three are used for data processing and structure determination. Unit-cell parameters within these data sets differ less than 2s. The accuracy of the unit-cell parameters from microED is expected to be highly influenced by local thermal expansion and crystal degradation due to the electron beam. Merging unit-cell parameters of multiple crystallites is expected to improve the precision significantly but the accuracy will be only marginally influenced due to the above-mentioned effects.

S3. Fourier difference maps showing hydrogen positions

The following figures were made with the SHELX software and depict Fourier difference maps showing positive electron density (green) assigned to hydrogen atoms and the calculated hydrogen positions (white spheres).

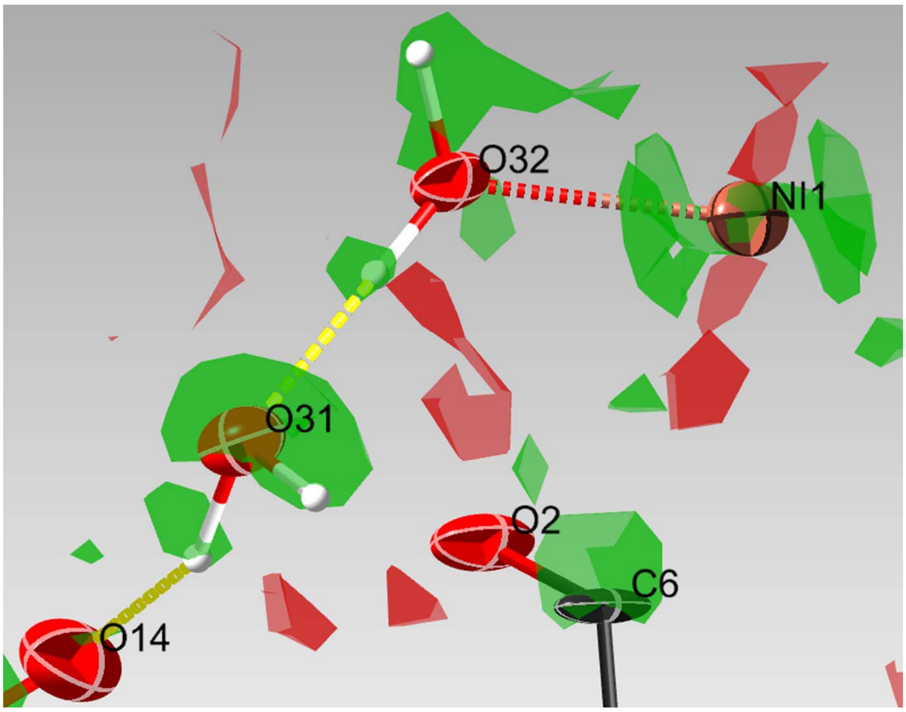

Figure S8 Fourier difference map showing the hydrogen positions and positive electron density (green) around O31 and O32.

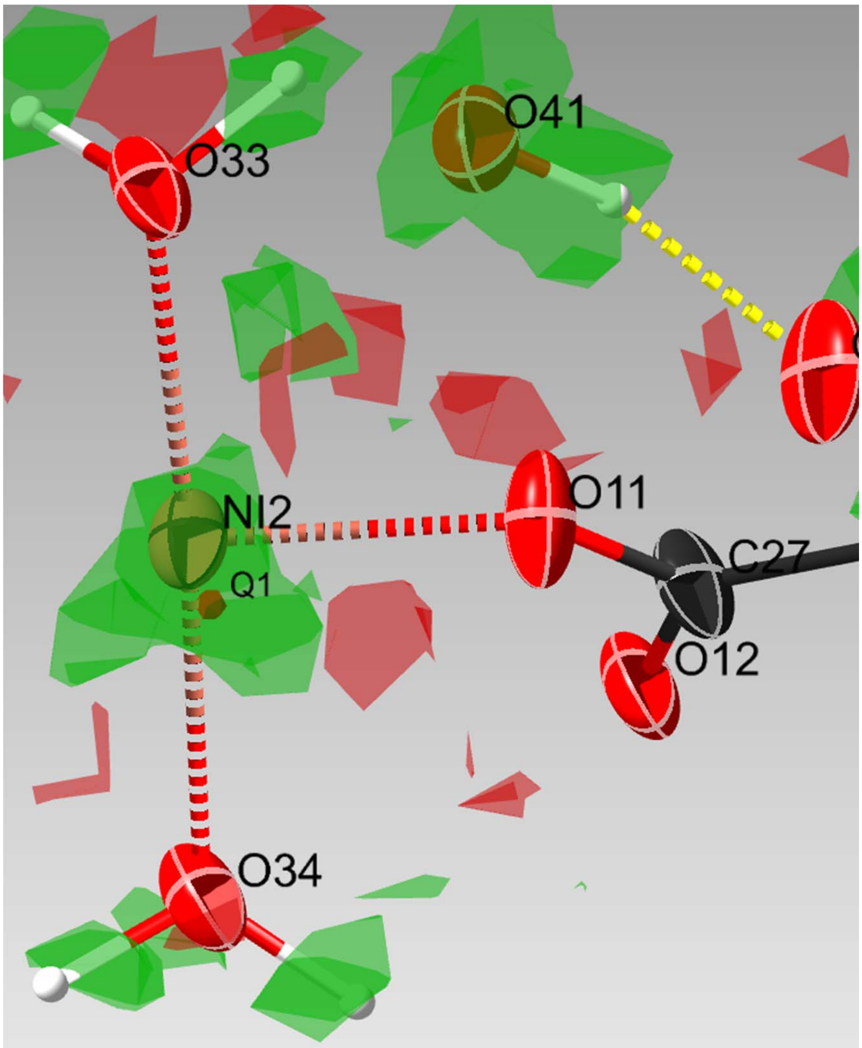

Figure S9 Fourier difference map showing the hydrogen positions and positive electron density (green) around O33, O34 and O41.

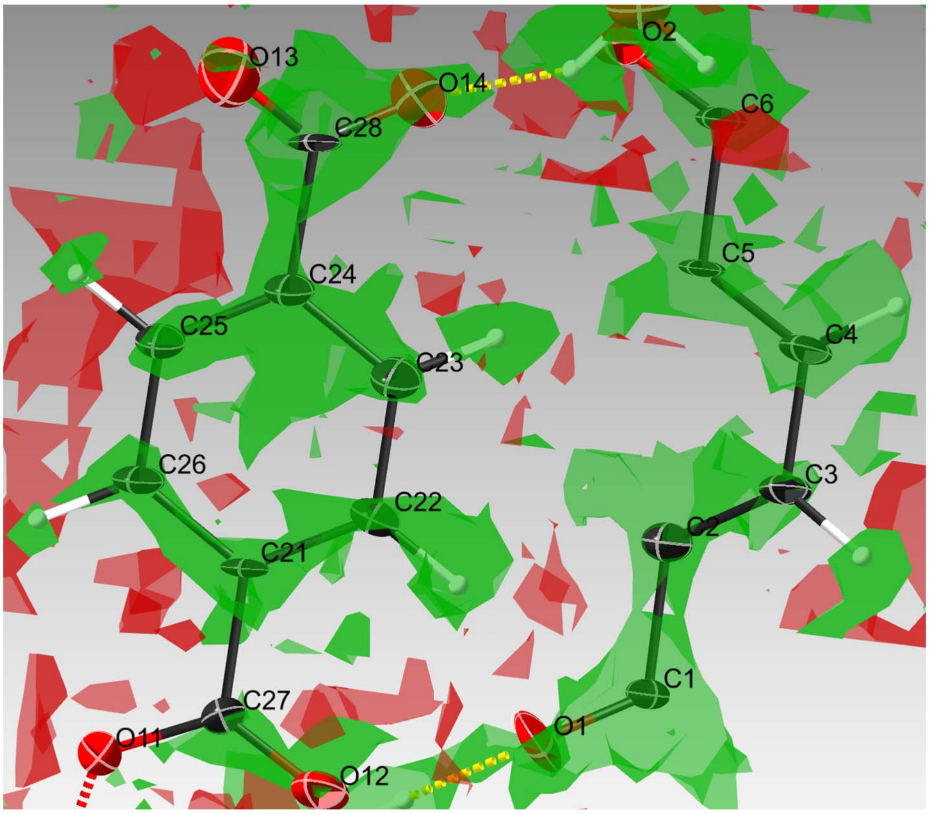

Figure S10 Fourier difference map showing the hydrogen positions and positive electron density (green) around O2, C22, C23, C25, C26, C3, and C4.

S4. Comparison of the crystal structures of TAF-CNU-1 (I) and Cu(BDT).3H_2O

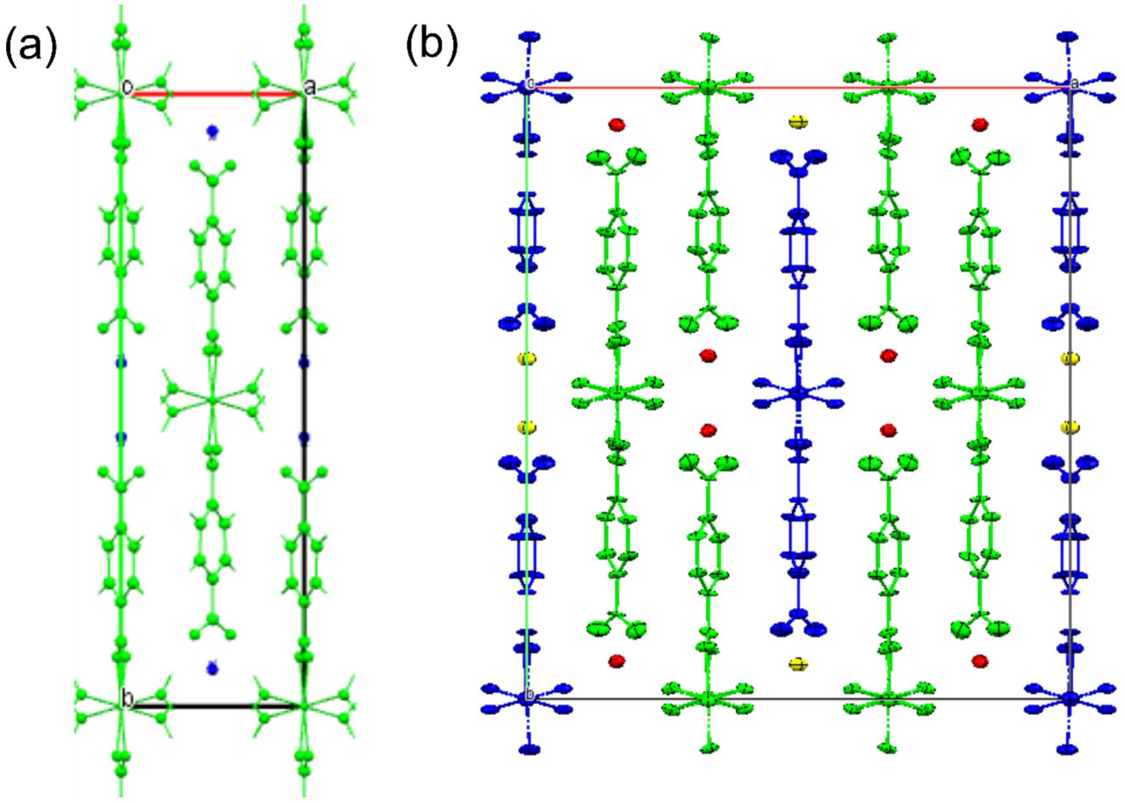

Figure S11 (a) View of the crystal structure of Cu(BDT).3H_2O (Cueto *et al*., 1991) along the *c*-axis direction. Molecular fragments are coloured green or blue by symmetry equivalence. (b) The crystal structure of TAF-CNU-1 (**I**) viewed also along the *c* axis, showing an approximately triplicated *a* axis. Fragments are also coloured by symmetry equivalence.

S5. Thermogravimetric Analysis

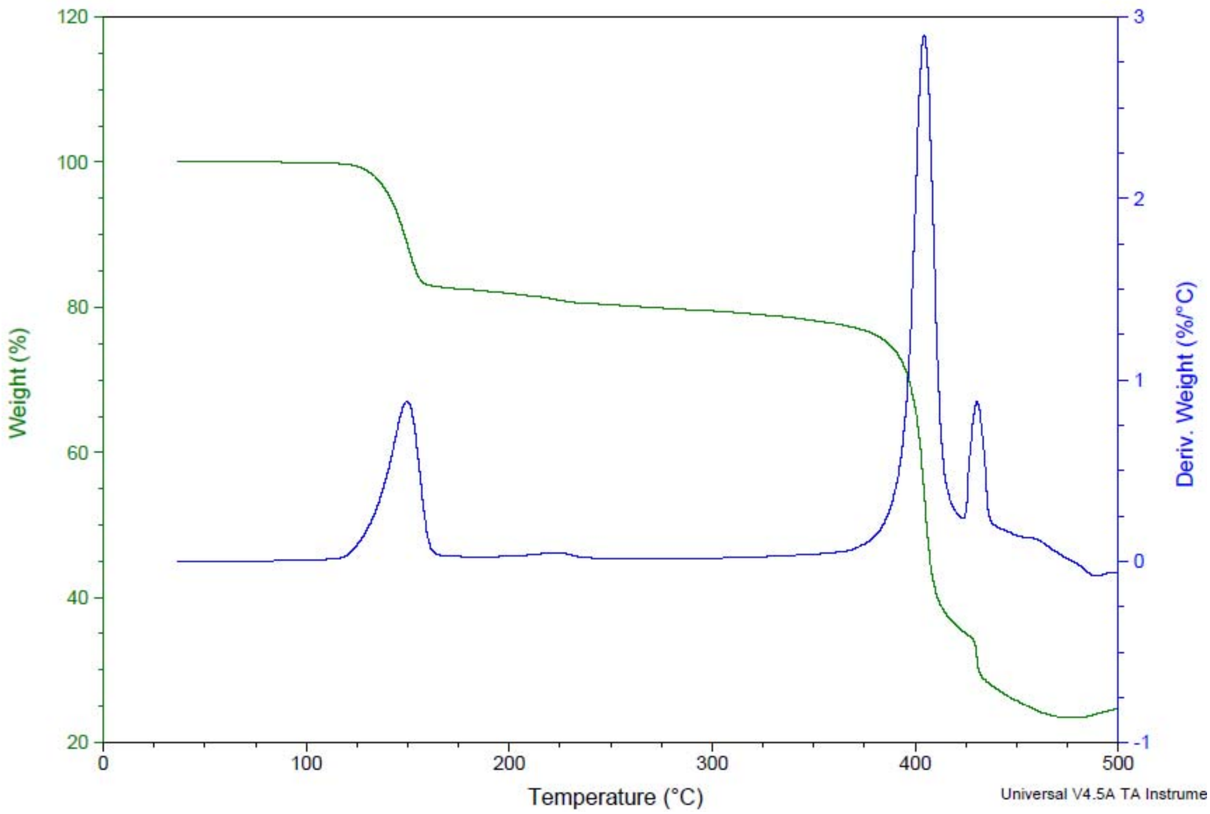

Figure S12 Thermogram of TAF-CNU-1 from room temperature up to 500°C (green) and its derivative (blue). Dehydration thermal events occur below 250°C. The thermal decomposition temperature for TAF-CNU-1 is around 400°C. A second decomposition step with around a quarter of the mass loss of the first event occurs at ~430°C. Both are observed as peaks in the weight derivative function (in blue).

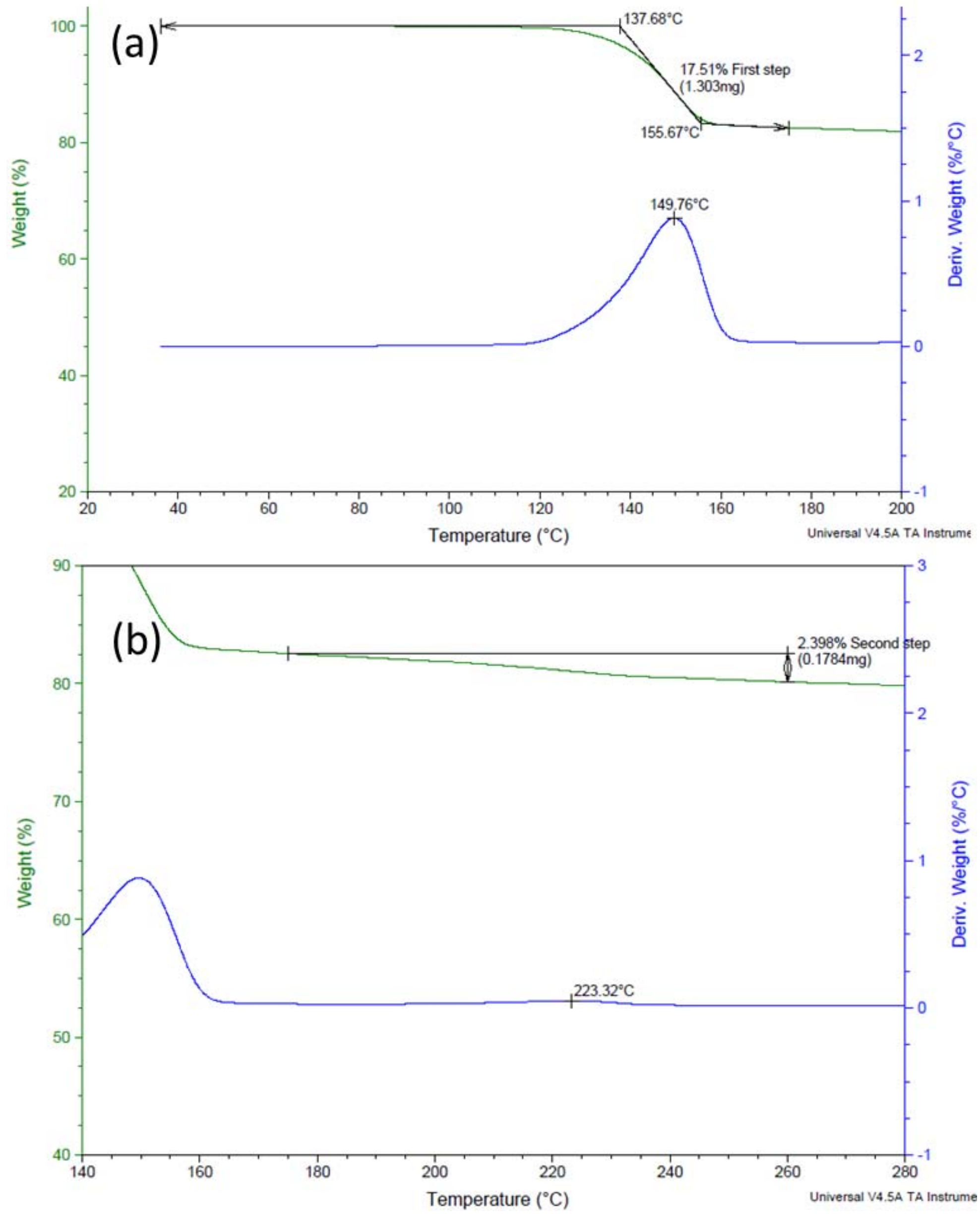

Figure S13 Thermogravimetric mass loss (green line) and its derivative (blue line) for TAF-CNU-1. (a) The first mass loss step of 17.51 % at 149.76°C. (b) The second mass loss step of 2.40% with maximum in the weight derivative at around 223°C. The total mass loss by thermogravimetry is 19.9%, corresponding to a trihydrate. The complete thermogram is shown in Figure S12.

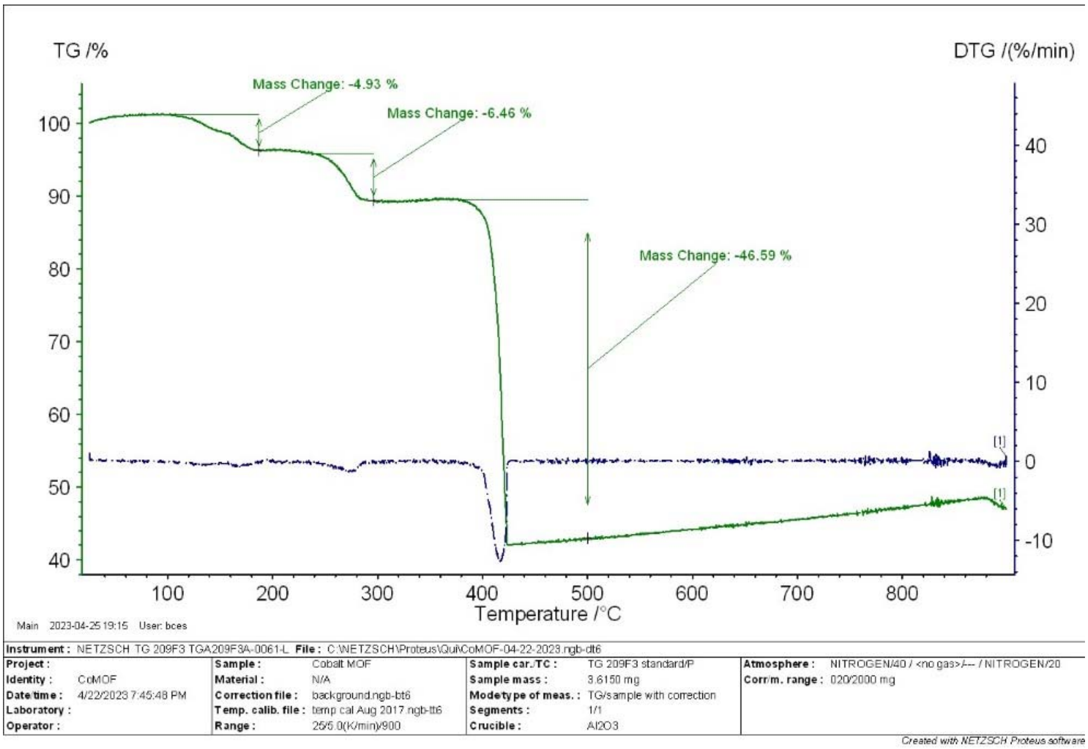

Figure S14. Thermogravimetric curve (green) and its derivative (blue) for **II**.

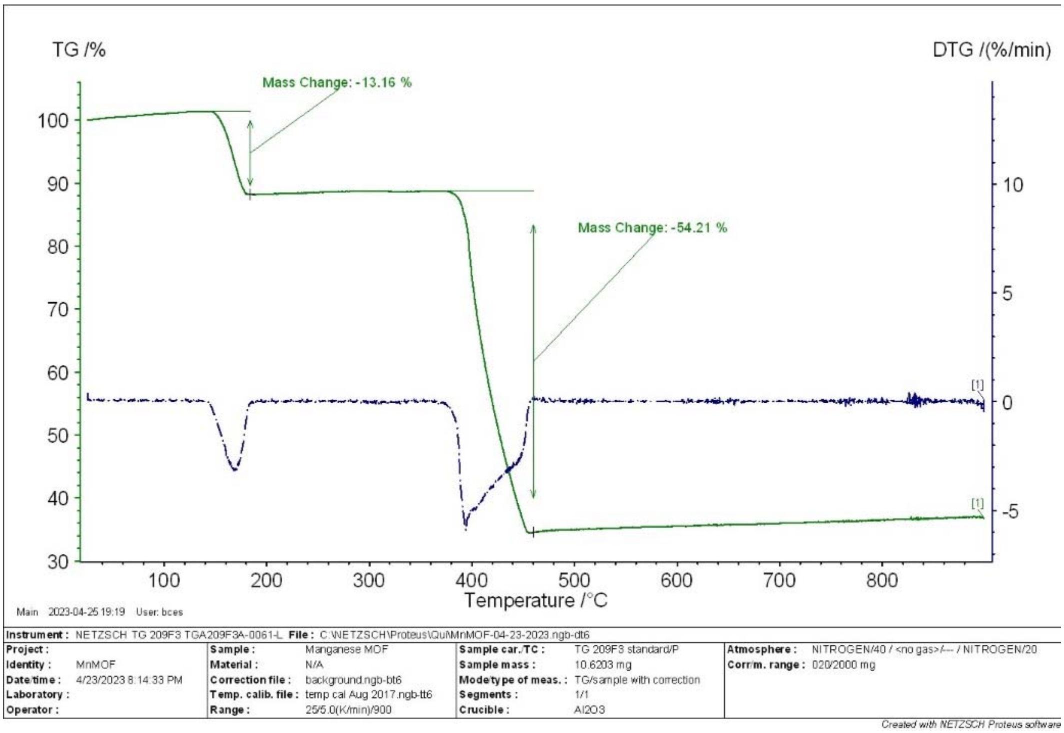

Figure S15. Thermogravimetric curve (green) and its derivative (blue) for **III**.

S6. Scanning electron microscopy

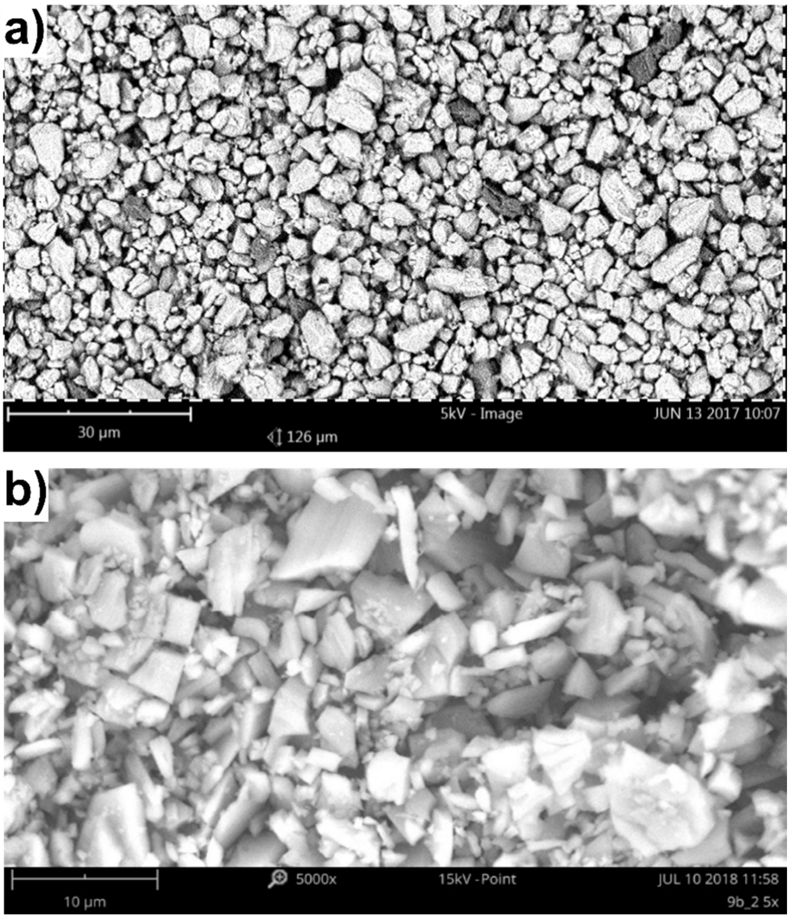

Figure S16. SEM micrographs of (a) **II** and (b) **III**.

References

Bruker (2016). *SADABS*, version 2016/2. Bruker AXS Inc., Madison, Wisconsin, USA.

Bruker (2019). *SAINT*, version 8.40B. Bruker AXS Inc., Madison, Wisconsin, USA.

Bruker (2022). *APEX* suite of crystallographic software. *APEX4*, version 2022.1-1. Bruker AXS Inc., Madison, Wisconsin, USA.

Doyle, P. A. & Turner, P. S. (1968). *Acta Cryst. A*24, 390–397.

Huang, Z., Grape, E. S., Li, J., Inge, A. K. & Zou, X. (2021). *Coord. Chem. Rev*. **427**, 213583.

Hübschle, C. B., Sheldrick, G. M. & Dittrich, B. (2011). *J. Appl. Cryst.* **44**, 1281–1284.

Palatinus, L., Brázda, P., Jelínek, M., Hrdá, J., Steciuk, G. & Klementová, M. (2019). *Acta Cryst.* B**75**, 512–522.

Petříček, V., Dušek, M. & Palatinus, L. (2014). *Z. Kristallogr.* **229**, 345-352.

Sheldrick, G. M. (2015). *Acta Cryst.* C**71**, 3–8.
